# Transcriptional Reactivation of Lignin Biosynthesis for the Heterologous Production of Etoposide Aglycone in *Nicotiana benthamiana*

**DOI:** 10.1021/acssynbio.2c00289

**Published:** 2022-09-19

**Authors:** Stacie S. Kim, Diego L. Wengier, Carin J. Ragland, Elizabeth S. Sattely

**Affiliations:** † Department of Chemical Engineering, Stanford University, Stanford, California 94305, United States; ‡ Department of Biology, Stanford University, Stanford, California 94305, United States; § Howard Hughes Medical Institute, Stanford University, Stanford, California 94305, United States

**Keywords:** plant metabolic engineering, transcriptional activation, lignin biosynthesis, heterologous biosynthesis, plant chassis, etoposide biosynthesis

## Abstract

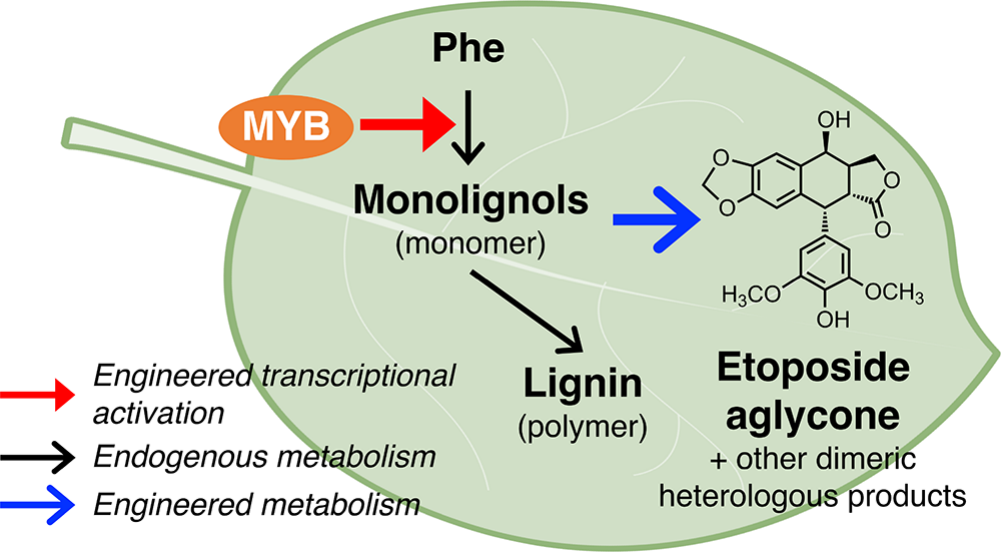

*Nicotiana benthamiana* is a valuable plant chassis for heterologous production of medicinal plant natural products. This host is well suited for the processing of organelle-localized plant enzymes, and the conservation of the primary metabolism across the plant kingdom often provides required plant-specific precursor molecules that feed a given pathway. Despite this commonality in metabolism, limited precursor supply and/or competing host pathways can interfere with yields of heterologous products. Here, we use transient transcriptional reprogramming of endogenous *N. benthamiana* metabolism to drastically improve flux through the etoposide pathway derived from the medicinal plant *Podophyllum* spp. Specifically, coexpression of a single lignin-associated transcription factor, MYB85, with pathway genes results in unprecedented levels of heterologous product accumulation in *N. benthamiana* leaves: 1 mg/g dry weight (DW) of the etoposide aglycone, 35 mg/g DW (−)-deoxypodophyllotoxin, and 3.5 mg/g DW (−)-epipodophyllotoxin—up to two orders of magnitude above previously reported biosynthetic yields for the etoposide aglycone and eight times higher than what is observed for (−)-deoxypodophyllotoxin in the native medicinal plant. Unexpectedly, transient activation of lignin metabolism by transcription factor overexpression also reduces the production of undesired side products that likely result from competing *N. benthamiana* metabolism. Our work demonstrates that synthetic activation of lignin biosynthesis in leaf tissue is an effective strategy for optimizing the production of medicinal compounds derived from phenylpropanoid precursors in the plant chassis *N. benthamiana*. Furthermore, our results highlight the engineering value of MYB85, an early switch in lignin biosynthesis, for on-demand modulation of monolignol flux and support the role of MYB46 as a master regulator of lignin polymer deposition.

## Introduction

Plants possess an expansive repertoire of biosynthetic pathways to specialized metabolites with broad chemical diversity. Their structural complexity arises from the combination and modification of a relatively small set of primary metabolic building blocks such as amino acids, isoprenoid precursors, and acetyl-CoA, as well as plant-specific molecules including monolignols. The conservation of these core metabolic processes across the Plant Kingdom allows for the transfer of heterologous specialized plant metabolic pathways from difficult-to-cultivate medicinal plants into a plant chassis lab model such as *Nicotiana benthamiana*. However, because primary metabolism is a highly coordinated and controlled network that is optimized for concerted growth and development, it can limit flux through an engineered pathway.^1^ Yield optimization therefore may require redistribution of the native supply of primary metabolites into specialized metabolism ideally with minimal impacts on host viability. Successful approaches include overexpression of individual native enzymes that helped alleviate precursor supply bottleneck^2,3^ and the expression of orthogonal pathways that override native regulation.^4,5^

Tuning the endogenous transcription regulation of the plant host has been proposed as a potentially more efficient engineering strategy to feed a heterologous pathway, as it takes advantage of native coordination of primary metabolic genes.^6,7^ Transcription factors orchestrate the dynamic network of cellular responses to biotic and abiotic stimuli, as related to developmental stage, external stress, homeostasis, and natural metabolism.^8−10^ By directly binding to DNA or interacting with DNA-binding proteins, transcription factors control the metabolic network by up- or down-regulating gene expression levels. As more of the ∼2000 transcription factors found in a given plant species are functionally characterized, there has been interest in applying transcription regulation to metabolic engineering for various applications including reprogramming of differentiation in somatic cells^11^ and reactivation of endogenous metabolism.^12,13^

Changes in endogenous plant metabolism have been demonstrated with the use of transcription factors for a number of biosynthetic pathways such as anthocyanins (overexpression of snapdragon activators Del and Ros1 in tomato),^13^ isoprenoids (silencing of repressor *Ms*YABBY5),^14^ isoflavones (transgenic *Nicotiana tabacum* coexpressing *At*MYB12 and *Gm*IFS1),^15^ and lignin (transgenic *N. tabacum* expressing *Medicago* WRKYs).^12^ Given this precedence, we considered that reprogramming host metabolism with transcription factors could be an effective way to boost yields of a transiently expressed heterologous pathway and sought to test this hypothesis in the context of etoposide aglycone biosynthesis in *N. benthamiana*.

Etoposide is a clinically used chemotherapeutic and is produced from lignans found in the medicinal plant *Podophyllum* spp*.* The etoposide aglycone (EA)^2^ can be produced in *N. benthamiana* by *Agrobacterium*-mediated transient expression of pathway genes. However, yields are limited by the availability of coniferyl alcohol (CA), a monolignol that is also a main building block for the abundant plant biopolymer lignin. In our previous work,^3^ we showed that exogenous addition of CA increased yields of (−)-deoxypodophyllotoxin (DPT) in *N. benthamiana* leaves. By boosting CA production through overexpression of the canonical primary metabolic enzymes, we achieved milligram-level production of DPT.^3^ Despite these yield improvements, the levels of the lignan products were still low relative to what has been observed for lignin biosynthesis engineering (e.g., ∼30 mg/g in transgenic tobacco expressing *Medicago truncatula* transcription factors).^12^ In contrast, the high flux through the monolignol pathway required for generating large quantities of lignin suggested that the innate capacity in plant tissue for diverting fixed carbon to CA could be very high. Although CA is a ubiquitous plant metabolite, we reasoned that it is likely not present in leaves at the time of *Agrobacterium*-mediated DNA transfer given that (1) epidermal cells have already fully expanded, (2) lignin biosynthesis has already ceased, and (3) the leaf parenchyma does not normally contain high levels of lignin (e.g., compared to the sclerenchyma).

**Figure 1:**
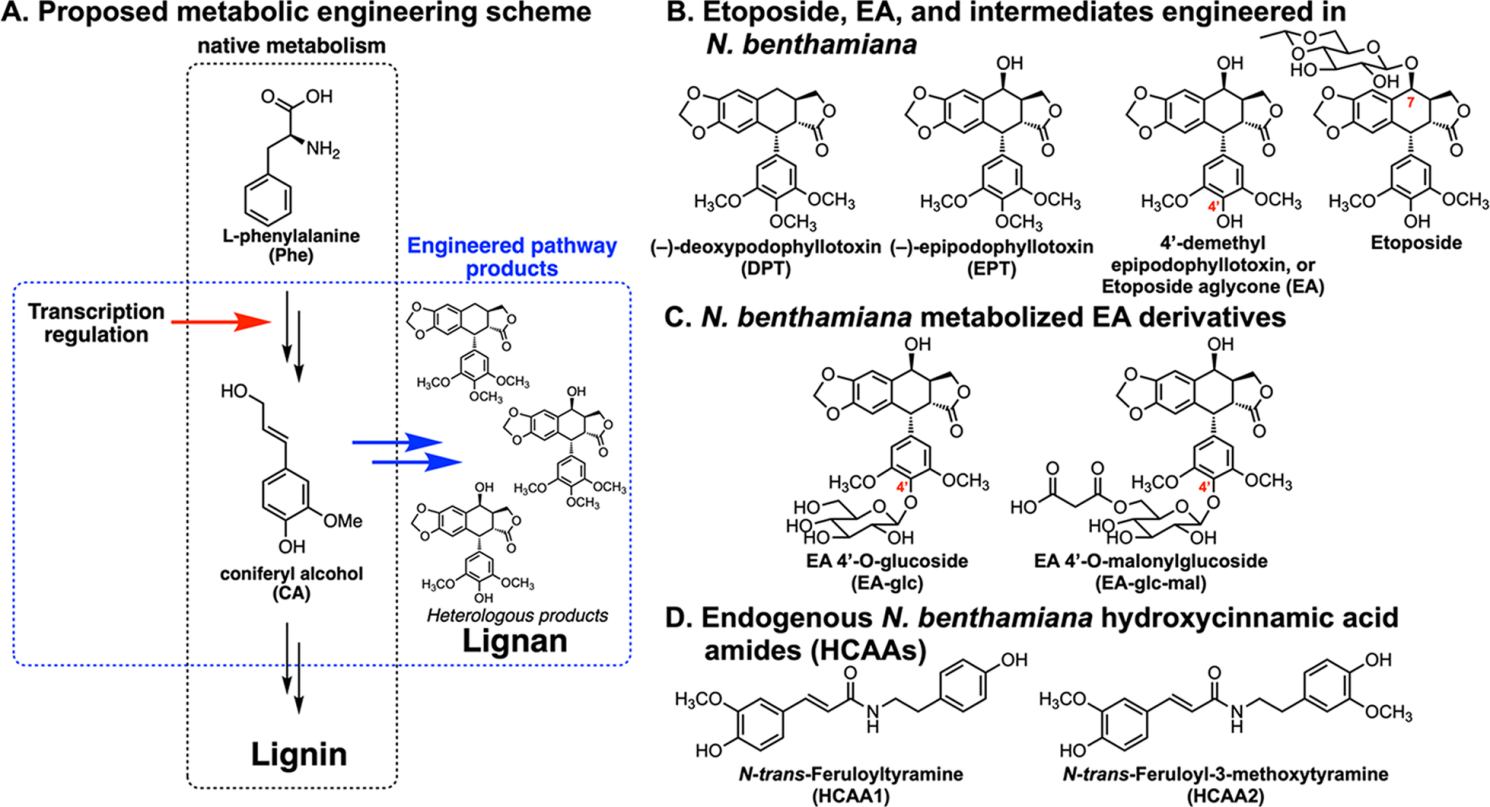
Proposed scheme for transcriptional regulation engineering and etoposide-related heterologous products. (A) Proposed transcriptional regulation engineering to divert native metabolism for high-value heterologous product biosynthesis. The red arrow represents activation, black arrows represent the native metabolic pathway, and blue arrows represent the heterologous pathway. (B–D) Chemical structures of desired heterologous products and other *N. benthamiana* endogenous metabolites and metabolic products. *O*-Glycosylation on C7 is a synthetic modification of EA to produce etoposide, while undesired 4′-*O*-glycosylation (shown in panel C) results from native metabolism in *N. benthamiana*.

In lignin biosynthesis, as related to development^16−18^ or defense response,^19,20^ transcriptional regulation is linked not only to the biosynthesis of monolignols—building blocks of lignin—but also other components of the secondary cell wall such as cellulose and xylan.^21−23^ Transcription factors involved in lignin biosynthesis are often identified with their ability to bind to the AC elements, common regulatory elements present in the promoter or 5′ untranslated regions of monolignol biosynthetic genes.^24,25^ Among those, certain transcription factors, such as MYB58, MYB85, and MYB103, are believed to be direct switches to monolignol biosynthesis,^26,27^ while others, such as SND1, MYB46, and VND6/7 (specific to xylem vessel formation), are considered to be master switches involved in controlling expression levels of those direct transcription factors and multiple processes.^28−31^ While characterization of transcription factors involved in monolignol biosynthesis has often been signified by ectopic deposition of lignin or reporter activation under the control of the promoter of a monolignol biosynthetic gene (e.g., 4CL),^27^ a primary metabolite flux increase has not been directly observed, because these products are believed to be readily utilized by the upregulated downstream processes (e.g., monolignols undergoing dehydrogenative polymerization to form lignin or dimerization to form lignans). For this study, we sought to reactivate early lignin biosynthesis in leaf cells during the heterologous pathway expression to improve the yields of engineering medicinal lignans. We hypothesized that overexpression of lignin biosynthesis transcription factors would increase coniferyl alcohol availability, which would translate into increased production levels of etoposide aglycone and its intermediates (Figure 1A).

## Results and Discussion

### Repurposing of Lignin-Associated Transcription Factors for Lignan Biosynthesis

With ∼2000 transcription factors (TFs) identified in the *Arabidopsis thaliana* genome^32−35^ and a wealth of genetic and functional characterization of those TFs available,^36,37^ we chose to focus on heterologous expression of *A. thaliana* TFs in *N. benthamiana* using *Agrobacterium*-mediated transient DNA delivery for engineering the transcriptional regulation of lignan biosynthesis. Six TFs previously characterized and associated with lignin and monolignol biosynthesis were selected for initial study: *At*MYB85,^26^*At*MYB46,^28^*At*MYB103,^26^*At*MYB58,^27^*At*MYB63,^27^ and *At*VND6.^30,31^ Previously, we have found that individual overexpression of each CA pathway enzyme with DPT biosynthetic enzymes resulted in a yield of 4.3 mg/g dry weight (DW) for the lignan DPT,^3^ comparable to the biosynthetic yield of 4.5 mg/g DW found in the roots of the native plant *Podophyllum hexandrum*.^38^ We sought to test if expression of a single TF alone instead could further improve pathway flux beyond the precedent.

**Figure 2:**
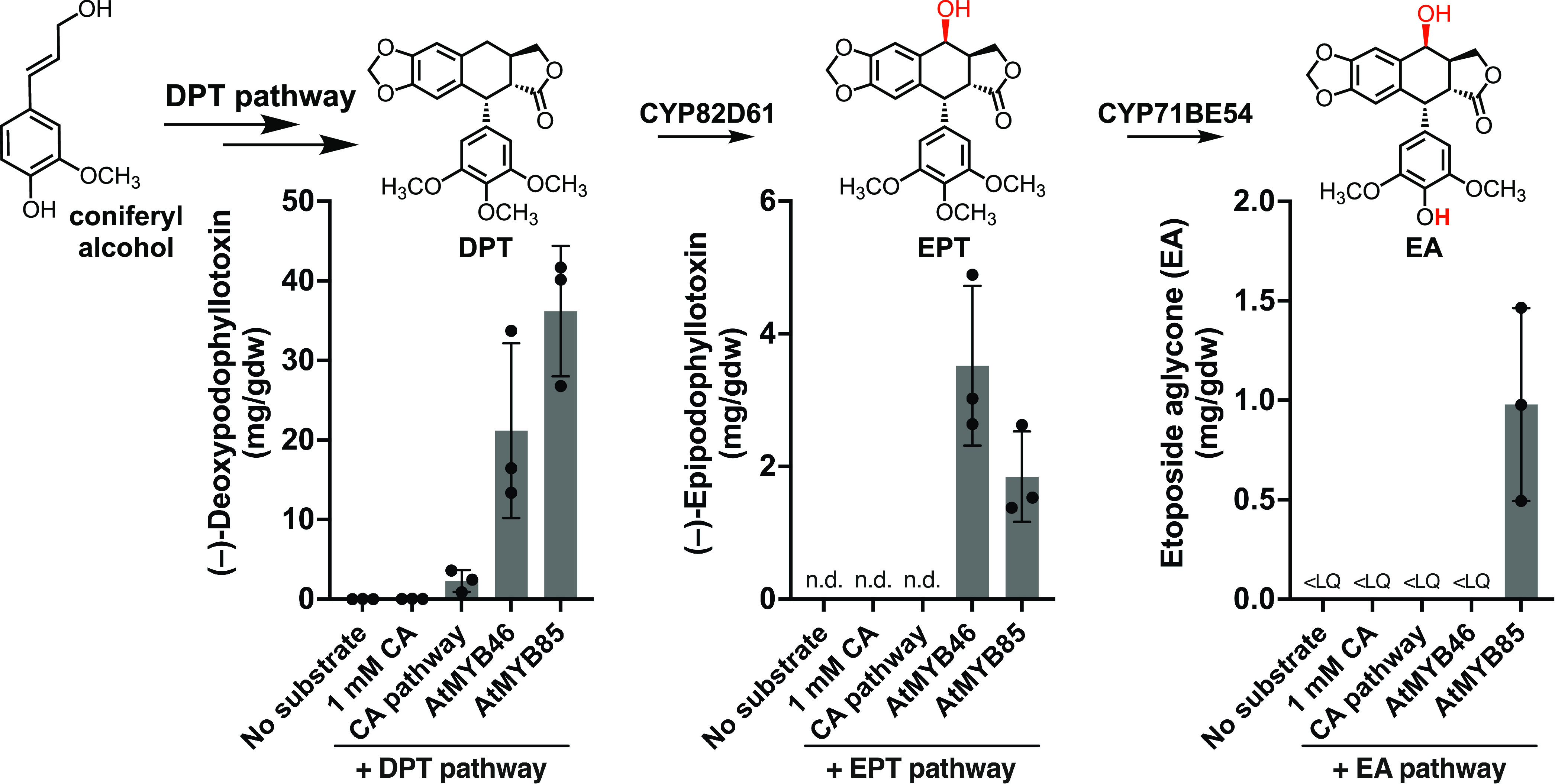
Biosynthetic yields of etoposide intermediates 7 days post infiltration promoted by *Agrobacterium*-mediated transient expression of CA pathway genes or single lignin-associated TFs in *N. benthamiana* leaves. DPT pathway: dirigent protein (*Ph*DIR), pinoresinol-lariciresinol reductase (*Ph*PLR), secoisolariciresinol dehydrogenase (*Ph*SDH), *Ph*CYP719A23, *Ph*OMT3, *Ph*CYP71CU1, *Ph*OMT1, and *Ph*2ODD; EPT pathway: DPT pathway and *Ph*CYP82D61; EA pathway: EPT pathway and *Ph*CYP71BE54; No substrate: no exogenous addition of the substrate CA; 1 mM CA: exogenous addition of CA; CA pathway: eight canonical biosynthetic enzymes converting phenylalanine to coniferyl alcohol;^39,40^ n.d.: no data; <LQ: below limit of quantification (<0.04 mg/g DW). Biosynthetic yields were quantified based on EIC peak integrations (*m*/*z* of [M + H]^+^) as detected by LC–MS in comparison to standard curves. Bar heights show the means of biological triplicates, and error bars show standard deviations.

When each transcription factor was coexpressed with the DPT biosynthetic pathway enzymes via *Agrobacterium*-mediated transient expression, *At*MYB46 and *At*MYB85 promoted the highest yields for DPT production (Figure 2 and Figure S1). Moreover, with CYP82D61 converting DPT to EPT, milligram-scale yields were obtained with either *At*MYB46 or *At*MYB85, up to 60-fold improvement from the biosynthetic yield of 57.8 μg/g DW achieved with exogenous addition of the precursor (+)-pinoresinol.^2^ Finally, with both CYP82D61 and CYP71BE54 present to convert DPT to EA, *At*MYB85 coexpression resulted in a striking improvement in EA biosynthetic yield at 0.98 mg/g DW, a 95-fold increase from the precursor-supplied biosynthetic yield of 10.3 μg/g DW.^2^ Interestingly, we observed significant accumulation of intermediates only when *At*MYB85 was expressed (Figure S2). The accumulation of intermediate compounds in the biosynthetic pathway indicated that the precursor supply increase induced by *At*MYB85 surpassed the capacity of pathway throughput by overexpression of the transgenes. That is, with *At*MYB85 reactivation of monolignol biosynthesis, the precursor supply level was no longer limiting. Additionally, while DPT consumption was noted in samples expressing the full EA pathway with *At*MYB46 or *At*MYB85, EA-associated metabolite peaks were among the most enriched mass features in the *At*MYB85-expressing samples, but not with *At*MYB46 (Figure S2C,D). These data suggest that other pathways induced by *At*MYB46 may compete with EA production.

Given the marked yield improvement of late-stage etoposide intermediates by either *At*MYB46 or *At*MYB85, we next coexpressed the EA pathway with both *At*MYB46 and *At*MYB85 simultaneously to determine if there is a synergistic effect. Unexpectedly, the EA yield with both transcription factors present was significantly lower than that with *At*MYB85 (Figure S3). These data suggest that the effect of *At*MYB46 overexpression overrides shifts in metabolism caused by overexpression of *At*MYB85 alone. Given that *At*MYB46 is known to regulate downstream expression of *At*MYB85 in *Arabidopsis*,^28^ it is possible that *At*MYB46 regulates a larger portion of metabolism than *At*MYB85, e.g., whereas *At*MYB85 seems to regulate the production of lignin precursors, *At*MYB46 could also control pathways that result in further metabolism of these precursors and the biosynthetic intermediates, channeling phenylpropanoid monomers toward lignin and limiting their availability for the etoposide aglycone pathway. Furthermore, we questioned whether endogenous copies of the transcription factors would activate the desired endogenous metabolism more effectively compared to their homologs from *Arabidopsis*. Interestingly, overexpression of *N. benthamiana* MYB46 and MYB85 did not boost EA production to the same extent that the *Arabidopsis* versions could despite their high degrees of sequence similarity in the DNA-binding domain (Figure S4 and Figure S5). Accumulation of EA and its metabolites when the EA pathway was simultaneously overexpressed with *Nb*MYB85a or *Nb*MYB85b did not surpass the levels obtained with *At*MYB85 coexpression.

Several other parameters were tested in an attempt to optimize product yields. First, we examined the effect of introducing less *At*MYB85-harboring *Agrobacterium* strain with respect to the *Agrobacterium* strains harboring the EA pathway genes. Overall, we found little difference in EA yields in the range of 0.03–0.1 OD for inoculation, but we did notice a continued decrease in the EA glycoside production with increasing inoculum levels of the *At*MYB85-harboring *Agrobacterium* strain (Figure S6A,B,D). In a separate experiment, we tested the length of time prior to collection of leaf tissue and found that yields from leaves extracted 5–9 days post infiltration (dpi) were typically higher than those from leaves extracted 13–18 dpi (Figure S6C,E).

### Suppression of Undesired Glycosylation in *At*MYB85-Expressing *N. benthamiana* Leaves

**Figure 3:**
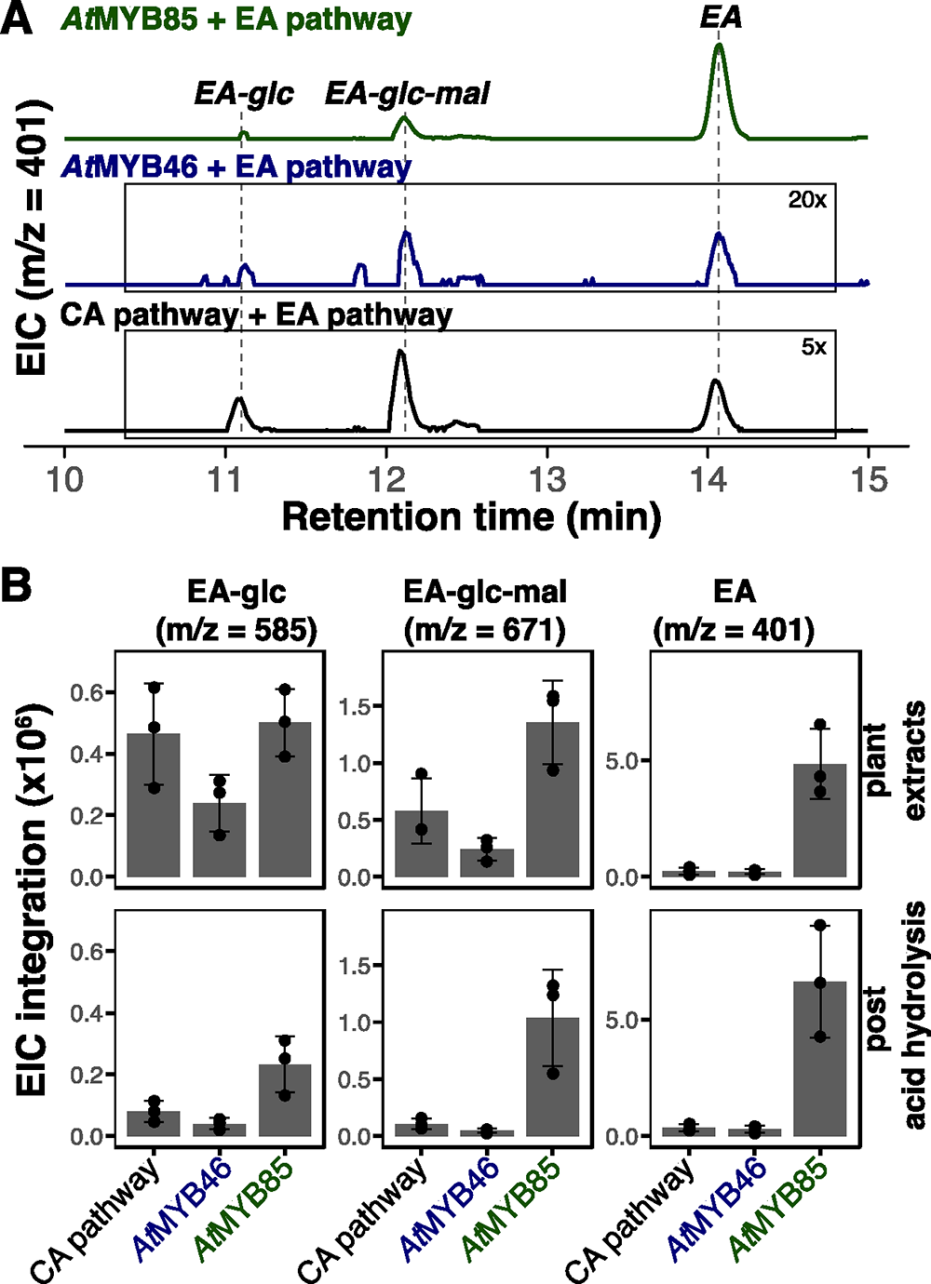
Impact on the EA 4′-*O*-glycosylation by *At*MYB85 coexpression. (A) Extracted ion chromatograms (EIC) for EA (*m*/*z* [M + H]^+^: 401.1231) as detected by LC–MS in *N. benthamiana* leaves expressing the EA pathway along with the CA pathway, *At*MYB46 or *At*MYB85. Earlier eluting peaks are in-source fragmentation products of EA 4′-*O*-glycosides. The *At*MYB85 chromatogram is to scale, and the *At*MYB46 chromatogram is scaled by 20× and the CA pathway by 5×. (B) EA and its metabolic products (4′-*O*-glycosides) in plant extracts from *N. benthamiana* expressing the heterologous EA pathway. The *x*-axis shows the source of CA biosynthesis boosted by the CA pathway, *At*MYB46, or *At*MYB85 coexpression. Acid-hydrolysis treatment removes glycosyl groups from EA 4′-*O*-glycoside products. Data points show EIC peak integrations for corresponding ion species (*m*/*z*: 585.1579 [M + Na]^+^ for EA-glc, 671.1583 [M + Na]^+^ for EA-glc-mal, and 401.1231 [M + H]^+^ for EA) as detected by LC–MS. Bar heights indicate the means of biological triplicates, and error bars indicate standard deviations.

In the course of our work, we noted that expression of *At*MYB85 and *At*MYB46 resulted in similar product yields for the DPT and EPT pathways, but that of *At*MYB85 was uniquely effective in boosting the yield of EA when coexpressed with the EA pathway genes. To probe this result further, we analyzed the metabolites made after expression of the EA pathway *in planta* (Figure 3A). Previously, we found that EA can be further metabolized in *N. benthamiana* leaves by endogenous enzymes, reducing the desired product (EA) yield due to formation of the 4′-*O*-glycosylation metabolites: EA 4′-*O*-glucoside and EA 4′-*O*-malonylglucoside (see Figure 1C).^2^ To our surprise, not only did the EA yield improve significantly in the *At*MYB85-expressing samples, but the ratios of EA and the 4′-*O*-glycosides had been altered relative to coexpression with MYB46 or CA pathway genes (Figure 3B). Even after adding an acid-hydrolysis step to remove glycosyl groups from EA, the net quantity of EA 4′-*O*-aglycone produced in leaves still remained the highest in the *At*MYB85-expressing samples. Thus, the striking improvement in EA biosynthetic yields promoted by *At*MYB85 overexpression may be attributed to the combination of two factors: (1) increased precursor (CA) availability in the *N. benthamiana* leaves funneled into EA biosynthesis and (2) the apparent suppression of undesired endogenous metabolism of EA by *At*MYB85 relative to *At*MYB46. In a separate effort, we also attempted to identify an endogenous glycosyltransferase responsible for the undesired metabolism but were unable to find a unique *N. benthamiana* enzyme (see Supplementary Discussion).

### Optimal CA Biosynthesis As Regulated by *At*MYB85 in *N. benthamiana* Leaves

In our previous work,^3^ we observed the highest level of DPT accumulation only when all eight primary metabolic genes from *P. hexandrum* that are part of the CA biosynthetic pathway (PAL, C4H, 4CL, HCT, C3H, CCoA-OMT, CCR, and CAD) were overexpressed in *N. benthamiana*, and omission of even a single gene resulted in at least a half-fold reduction in yield. Since overexpression of *At*MYB85 resulted in DPT accumulation at higher levels than the CA pathway overexpression, we reasoned that *At*MYB85-coordinated control of the CA pathway was beneficial for increasing the precursor availability. Although MYB85 is known to regulate early lignin biosynthesis, the direct induction of CA pathway genes has not been measured. Thus, we measured transcript levels of these enzymes in *N. benthamiana* leaves expressing GFP (control) or *At*MYB85 with EA pathway enzymes by qRT-PCR. We confirmed that all eight enzymes involved in the CA pathway were upregulated by at least ∼4-fold compared to the GFP control (Figure S7). Given that transcription factor overexpression typically results in higher yields than the CA pathway overexpression, we speculate that the transcription factor control may better reflect the endogenous expression ratios for each gene of the pathway, ensuring optimal levels for proper CA biosynthesis—as opposed to individual gene overexpression at the saturating levels. In one possibility, unregulated gene expression could result in the feedback-inhibition mechanism of pathway intermediates, affecting enzyme activity upstream or downstream—a prominent phenomenon found in the monolignol biosynthesis.^41−46^

### Reactivation of Lignin Biosynthesis in *N. benthamiana* Leaves by *At*MYB46

**Figure 4:**
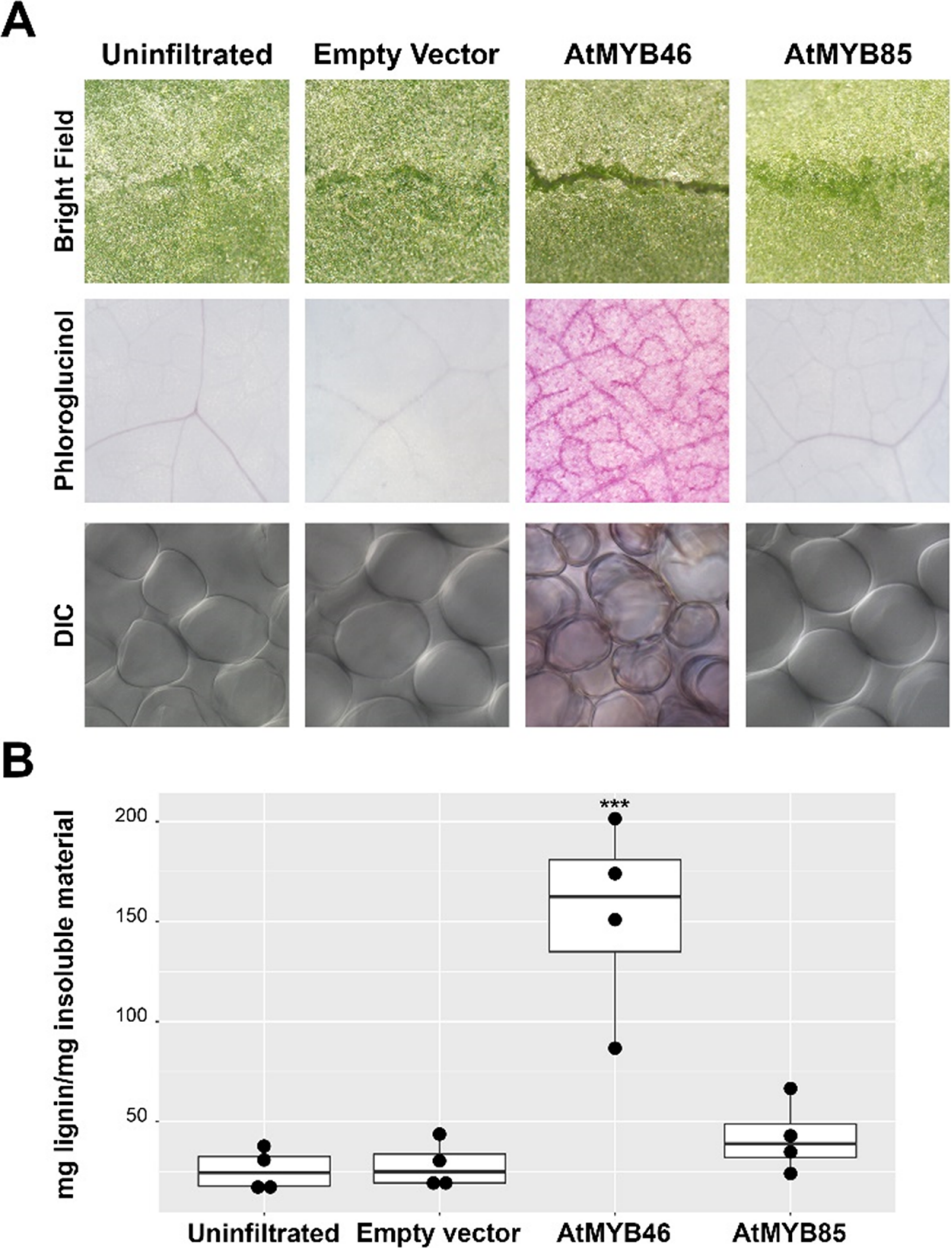
Reactivation of lignin biosynthesis in *N. benthamiana*. (A) Bright field images of uninfiltrated and infiltrated leaves show that *At*MYB46-expressing leaves break upon folding. Phloroglucinol stain (magenta) reveals extensive lignin deposition in *At*MYB46-expressing leaves, in parallel with xylem-like lignin banding in cell walls of mesophyll cells as observed by DIC microscopy. Magnifications: bright field and phloroglucinol stain, 2×; DIC, 400×. (B) Lignin content as quantified by the acetyl bromide method. Data points show the means of triplicates from four different experiments (*n* = 4), and error bars indicate standard deviations. ANOVA on log-transformed data showed a statistical difference between means (***p < 0.0001). Multiple comparisons showed that lignin content in *At*MYB46-expressing leaves was the only value statistically different from the rest.

While both *At*MYB46 and *At*MYB85 are involved in lignin biosynthesis activation, *At*MYB46 is considered to be a master switch controlling other transcription regulation mechanisms,^28,47^ and *At*MYB85 is thought to directly activate monolignol biosynthesis.^26^ Given the different yields obtained for DPT and EA production using these TFs, we sought to probe the differences in the mechanism of lignin biosynthesis reactivation induced by *At*MYB46 or *At*MYB85. Corroborating previous reports, overexpression of *At*MYB46 in the *N. benthamiana* leaves resulted in higher accumulation of lignin compared to the empty-vector control (Figure 4). In particular, *At*MYB46 resulted in a striking level of lignin accumulation with the leaves turning brittle 5–7 days post infiltration (Video S1 and Figure 4A). At the cellular level, overexpression of *At*MYB46, but not *At*MYB85, recapitulated lignin deposition, typically found in tracheary elements. It is noteworthy that *At*MYB85-overexpressing leaves did not react to the phloroglucinol stain, unlike the *At*MYB46-overexpressing samples, suggesting that lignin deposition proceeded to completion in the latter to a higher degree as previously reported in the literature.^48^ The differences in phenotypical outputs, both in cell wall deposition and physical properties, prompted us to investigate other metabolic signatures that could differentiate the roles of *At*MYB46 and *At*MYB85. We conducted untargeted metabolomic analysis and found that the metabolic profiles of *N. benthamiana* leaves expressing GFP, *At*MYB85 + EA pathway or *At*MYB46 + EA pathway were considerably different based on the principal component analysis (PCA) clustering (Figure S8). Of note, many of the mass features (metabolite signals defined by the observed *m*/*z* and retention time on reverse-phase chromatography) with significant PCA loadings for the *At*MYB46-expressing leaf tissue samples appeared to be of higher molecular weights compared to mass features in *At*MYB85-expressing leaf samples, which may be indicative of lignin polymerization in *At*MYB46-expressing leaves. In contrast, *At*MYB85 activated the biosynthesis of other small molecules (namely, hydroxycinnamic acid amides, or HCAAs, see Figure 1D) and may be involved in activation of competing metabolism other than lignin polymerization (Tables S4–S7 and Figure S9).

## Conclusions

**Figure 5:**
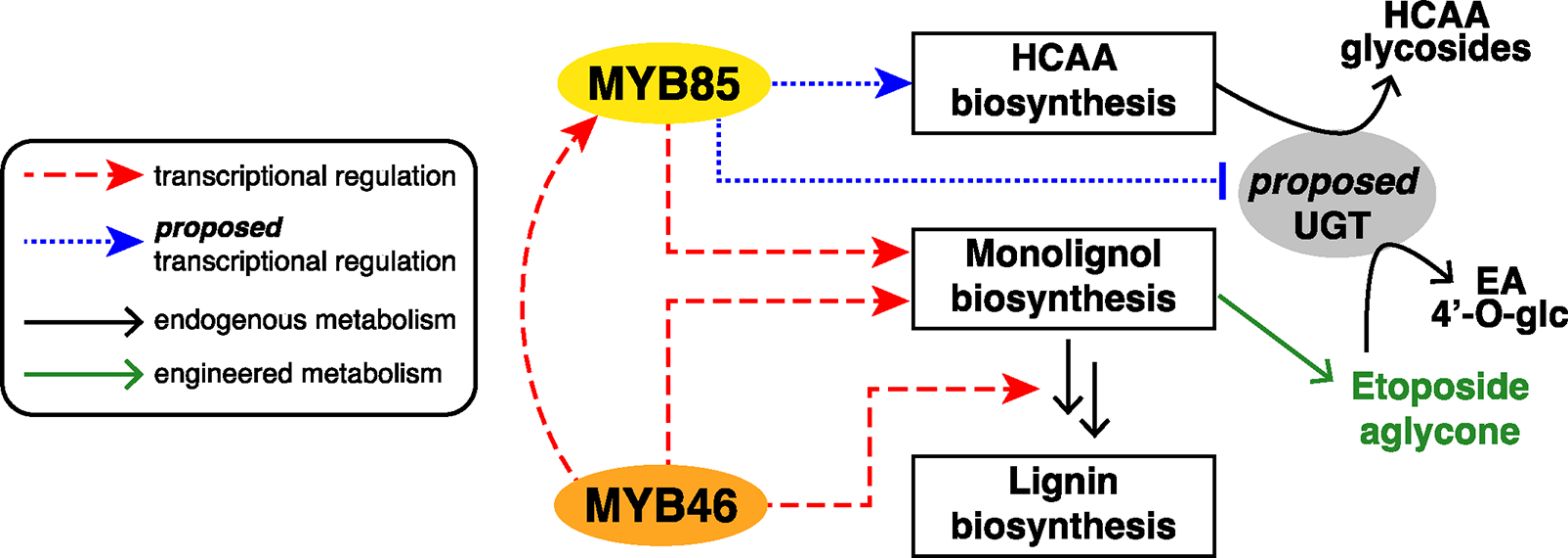
Model of transcription regulation engineering described in this study. Red dashed arrows indicate transcriptional regulation previously described in the literature, blue dotted arrows indicate proposed transcriptional regulation based on metabolite profiling, and black and green arrows indicate metabolite conversion.

Two hypotheses were considered to explain the unexpected suppression of EA product glycosylation observed in experiments with *At*MYB85 overexpression: (1) *At*MYB85 induces the biosynthesis of endogenous hydroxycinnamic metabolites (e.g., HCAAs), which could serve as glycosyltransferase substrates, and might act as competitive inhibitors to etoposide aglycone intermediates for glycosylation, and (2) *At*MYB85 represses the expression of certain UDP-glycosyltransferases (UGTs) that exhibit off-target activity on the heterologous products. In line with the first hypothesis, in our optimization efforts, we observed biosynthetic yields of EA glycosides inversely correlated with the production of the HCAA side products (Figure S6A,C). For the second hypothesis, we identified *Nb*UGT73A24 that can glycosylate EA and its intermediates, but the expression level of the gene did not decrease with *At*MYB85 overexpression (see Supplementary Discussion). However, it is possible that *At*MYB85 regulates the expression of other, not yet identified, *Nb*UGTs that contribute to EA glycosylation. Alternatively, there might be more complex metabolic regulation in place, for example, as is observed in flavonol biosynthesis and glycosylation.^49^ Examples of TFs that differentially regulate two competing branches of metabolism^50^ include *A. thaliana* MYB TFs that activate lignin biosynthesis while inhibiting flavonoid biosynthesis, including MYB85.^51^ In conclusion, our data show that overexpression of *At*MYB85 increases the yield of EA, and two synergistic mechanisms are possible: direct increase in endogenous precursor supply and suppression of the competing metabolism (Figure 5).

Here, we demonstrate the value of transient transcription factor expression to improve plant specialized metabolite biosynthesis in a plant heterologous host. As more plant pathways become targets for metabolic engineering, this approach is likely to be a valuable and convenient tool for altering the base metabolism in the plant chassis and could be applied to enhance production of other common pathway precursors in addition to monolignols (e.g., aliphatic amines for alkaloid biosynthesis or isoprenes for complex terpene production). *At*MYB85 overexpression with the EA pathway enzymes led to the highest biosynthetic yields of etoposide aglycone observed at ∼1 mg/g DW. Previously, precursor supply was observed to be more limiting than enzyme activity in EA biosynthesis in *N. benthamiana*, as evidenced by no intermediate accumulation in the secondary metabolism. Quantification of EA pathway intermediates in *N. benthamiana* leaves indicates that precursor availability is no longer limiting. Our findings reported here show promise for application of synthetic transcription regulation for metabolic engineering of biosynthetic pathways in plants.

## Methods

### Safety Statement

Handling of acetyl bromide was entirely conducted inside a chemical fume hood. No other unexpected or unusually high safety hazards were encountered.

### Candidate Gene Selection and Cloning

#### General Procedure

Q5 High-Fidelity 2X Master Mix was used for all PCR amplification steps, except for colony PCR, for which Hot Start *Taq* 2X Master Mix was used instead. All enzymes used for cloning were purchased from New England BioLabs, unless otherwise noted. Oligonucleotide primers were purchased from Integrated DNA Technologies. Plasmid constructs were assembled in an isothermal DNA assembly reaction as described by Gibson et al.^52^ NEB 5-alpha Competent *Escherichia coli* cells were used for plasmid storage and isolation. Plasmid DNAs were isolated from the liquid cultures of *E. coli* using the ZR Plasmid Miniprep kit (Zymo Research). Isolated plasmids and PCR amplicons were confirmed for correct sequences by Sanger DNA sequencing performed by Elim Biopharm.

#### pEAQ-HT Constructs for *N. benthamiana* Expression

*E. coli* strains harboring *At*MYB85 (TAIR accession: AT4G22680; clone name: PYAT4G22680), *At*MYB46 (TAIR accession: AT5G12870; clone name: U16973), *At*MYB63 (TAIR accession: AT1G79180; clone name: PYAT1G79180), *At*MYB103 (TAIR accession: AT1G63910; clone name: PYAT1G63910), *At*MYB58 (TAIR accession: AT1G16490; clone name: DKLAT1G16490), and *At*VND6 (TAIR accession: AT5G62380; clone name: DQ056734) were purchased from Arabidopsis Biological Resource Center (ARBC). The pET28a construct for *Sr*UGT71E1 and the *N. benthamiana* cDNA templates for *N. benthamiana* genes (*Nb*MYB46a [NCBI GenBank accession: OP121090], *Nb*MYB46b [OP121091], *Nb*MYB85a [OP121092], and *Nb*MYB85b [OP121093]) served as templates. The full-length coding sequences (CDS) were PCR-amplified from the plasmids with corresponding primers (see Table S1) using Q5 High-Fidelity 2X Master Mix (NEB), Gibson-assembled into pEAQ-HT^53^ predigested with AgeI and XhoI, and then transformed into *E. coli* 5-alpha chemically competent cells. Sequence-validated pEAQ-HT constructs were transformed into *Agrobacterium tumefaciens* (GV3101:pMP90) using the freeze–thaw method.

### *Agrobacterium*-Mediated Transient Expression in *N. benthamiana*

*Agrobacterium* strains prepared as described above were grown on LB plates supplemented with 10 μg/mL gentamicin and 50 μg/mL kanamycin for 1–2 days. The cells were resuspended in LB media and centrifuged at 8000*g* for 5 min, after which the supernatant was discarded. The cells were induced in 500 μL of induction media (10 mM MES buffer at pH 5.6 with 10 mM MgCl_2_ and 150 μM acetosyringone) for 1–2 h at room temperature. The cell suspension was further diluted with the induction media to the desired inoculum level (OD_600_ = 0.2, unless otherwise noted) based on the measured optical density at 600 nm and infiltrated on the underside of the *N. benthamiana* leaves with a needleless 1 mL syringe. Plants were 4–5 weeks old at the time of infiltration, grown under a 16 h light cycle. Biological replicates consisted of leaves of different ages (based on the stemming location on the plant, counting from the bottom) from different *N. benthamiana* plants from the same batch, unless otherwise noted. For metabolite extraction, leaves were harvested on 5–7 dpi.

### Methanol Extraction and LCMS Analysis of Plant Leaf Extracts

*N. benthamiana* leaves were flash-frozen in liquid nitrogen and lyophilized to dryness. The dry samples were homogenized in a ball-mill homogenizer with 5 mm-diameter stainless steel beads at 25 Hz for 2 min. Twenty microliters of 80% (v/v) methanol/water was added per mg of dry sample, and the samples were refluxed at 65 °C for 10 min. The resulting plant extracts were filtered with 0.45 μm PTFE filters prior to LCMS injection.

For *N. benthamiana*-metabolized EA glycosides, acid hydrolysis was conducted as previously described with slight modifications.^3^ The incubation time at 95 °C was kept at 10 min instead of 2 h due to concerns about the aglycone product stability.

Methanolic extracts were analyzed by reversed-phase column chromatography on a coupled Agilent 6520 Accurate-Mass q-ToF ESI mass spectrometer using a 5 μm, 2 × 100 mm Gemini NX-C18 column (Phenomenex) with mobile phases of water with 0.1% formic acid (A) and acetonitrile with 0.1% formic acid (B). The chromatography was run at a flow rate of 0.4 mL/min with the following gradient: 0–1 min, 3% B; 1–21 min, 3–50% B; 21–22 min, 50–97% B; 22–27 min, 97% B; 27–28 min, 97–3% B; and 28–32 min, 3% B. MS parameters were as follows: mass range, 50–1700 *m*/*z*; drying gas, 300 °C and 11–12 L/min; nebulizer, 35 psig; capillary, 3500 V; fragmentor, 150 V; skimmer, 65 V; octopole 1RF Vpp, 750 V; 699.3 ms per spectrum. The eluent in the first minute of each run was discarded to avoid salt contamination in the mass spectrometer.

### Histochemical Analysis of Lignification in the *N. benthamiana* Leaves

Lignin deposition was visualized with phloroglucinol staining. Round discs were cut from *N. benthamiana* leaves, bleached in 12.5% v/v acetic acid in ethanol (2–3 h with decanting and replenished solution once), and stored in 70% ethanol in water until further processing. The discs were incubated in an ethanolic solution of 2% phloroglucinol for 10 min and then transferred to 6 N HCl solution. After ∼10 min of incubation, samples were recorded with a digital camera coupled to a stereo microscope when staining of leaves and veins was observed. For higher magnification microscopy, leaf discs were cleared with Hoyer’s solution^54^ and analyzed by differential interference contrast (DIC) microscopy on a Leica DM2500 microscope.

### Lignin Content Quantification

Lignin content in *N. benthamiana* leaves (WT [no agro-infiltration], or expressing EV, *At*MYB46, or *At*MYB85) was quantified using the acetyl bromide method as described by Lee et al.,^20^ with slight modifications. The leaves were flash-frozen in liquid nitrogen and lyophilized to dryness. The dried samples were homogenized in a ball-mill homogenizer (Retsch MM 400) at 25 Hz for 2 min with 5 mm-diameter stainless steel beads. The ground samples were washed serially with 70% ethanol, chloroform/methanol (1:1 v/v), and acetone. The washed cell wall materials were completely dried at 45 °C under positive air flow. Two hundred microliters of 25% acetyl bromide solution in acetic acid was added per mg of sample, and the samples were incubated at 70 °C for an hour with occasional inversion every 10 min. The samples were cooled on ice and centrifuged for 5 min at 16,000*g*. One hundred microliters of the supernatant (or 25% acetyl bromide solution in acetic acid as the blank) was transferred to a glass vial and combined serially with 400 μL of 2 M NaOH, 70 μL of 0.5 M hydroxylamine hydrochloride, and 430 μL of acetic acid. Two hundred microliters of the resulting solutions was transferred to each well in a UV-specific 96-well microplate, and absorbance was measured at 280 nm. Measured absorbance values were corrected by subtracting the absorbance of the blank (similar to absorbance measured on an empty well), and the percentage of acetyl bromide soluble lignin (%ABSL) was calculated by the Beer–Lambert law assuming a path length of 0.539 cm^55^ and an extinction coefficient for *N. benthamiana* of 23.077 g^–1^ cm^–1^.^56^

## Supporting Information Available

*N. benthamiana* leaf turning brittle 5–7 days post infiltration (MOV)
Supplementary materials and methods, supplementary discussion, supplementary figures and tables, supplementary spectra, and supplementary references (PDF)

## Supplementary Material

sb2c00289_si_001.mov
