# Supplementary material for: Transcriptional Reactivation of Lignin Biosynthesis for the Heterologous Production of Etoposide Aglycone in Nicotiana benthamiana
Source: ACS Synth Biol. 2022 Sep 19;11(10):3379–87. doi: 10.1021/acssynbio.2c00289 (PMC9594330; doi:10.1021/acssynbio.2c00289)
Supplement: Supplementary file 2 — sb2c00289_si_002.pdf [file sb2c00289_si_002.pdf]

Supporting Information

Transcription-Factor Reactivation of Phenylpropanoid Biosynthesis and Application to Heterologous Production of Etoposide Aglycone In *N. benthamiana*

Stacie S. Kim,[1] Diego L. Wengier,[1] Carin J. Ragland,[2] and Elizabeth S. Sattely[1,3]*

[1]Department of Chemical Engineering, Stanford University, Stanford, California, 94305, United States
[2]Department of Biology, Stanford University, Stanford, California, 94305, United States
[3]Howard Hughes Medical Institute, Stanford University, Stanford, California, 94305, United States

*Correspondence should be addressed to E.S.S. (sattely@stanford.edu)

Table of Contents

Supplementary Materials and Methods....................S3
General S3
Materials....................S3
Agro-infiltrated *N. benthamiana* RNA isolation, RNA-seq dataset preparation, and cDNA synthesis .S3
RNA-seq data analysis....................S4
N. benthamiana gene primer design and qRT-PCR S4
Cloning pET28a constructs for *Sr*UGT71BE1 and *Nb*UGT expression in *E. coli*....................S5
E. coli lysate screening assays for *Nb*UGT candidates....................S5
LC-MS data analysis S6
Clustering analyses on metabolomic and transcriptomic data....................S7
Preparative HPLC purification S7
Isolation of EA, HCAA1, and HCAA2 from *N. benthamiana* leaves....................S7
Synthetic procedures....................S8
Supplementary Discussion S9
Identification of *Nb*UGT73A24 activity on EA metabolites....................S9

Supplementary Figures and Tables **S10**

Table S1. Primers used for pET28a constructs S10

Table S2. Primers used for pEAQ-HT constructs S11

Table S3. Primers used for qPCR S12

Figure S1. (–)-deoxypodophyllotoxin production with TF co-expression 5 days post-infiltration S13

Figure S2. Accumulation of biosynthetic intermediates S14

Figure S3. No synergistic effect between *At*MYB46 and *At*MYB85 on EA production S16

Figure S4. Protein sequence alignment of *N. benthamiana* homologs to *A. thaliana* transcription factors S17

Figure S5. Biosynthesis of etoposide aglycone and side products promoted by *N. benthamiana* homologs of *At*MYB46 and *At*MYB85 .... S18

Figure S6. Optimization of EA biosynthetic yields S20

Figure S7. Relative expression levels of CA pathway genes in *At*MYB85-expressing *N. benthamiana* leaves S21

Figure S8. PCA and hierarchical clustering of *N. benthamiana* leaves based on metabolite composition S22

Table S4. Metabolite peaks enriched in leaves expressing *At*MYB85 and EA pathway compared to those expressing GFP S23

Table S5. Metabolite peaks enriched in leaves expressing *At*MYB46 and EA pathway compared to those expressing GFP S24

Table S6. Metabolite peaks enriched in leaves expressing AtMYB85 compared to AtMYB46 expressing leaves S25

Table S7. Metabolite peaks enriched in leaves expressing *At*MYB46 compared to *At*MYB85 expressing leaves S26

Figure S9. MS/MS fragmentation analysis of HCAA-like metabolites enriched in *At*MYB85-expressing samples S27

Figure S10. Identification of *Nb*UGT73A24 responsible for host glycosylation activity S28

Figure S11. Relative expression levels of UGT homologs in *At*MYB85-expressing *N. benthamiana* leaves S30

Supplementary Spectra **S31**

Spectrum S1. ^{1}H NMR spectrum of isolated etoposide aglycone from *N. benthamiana* leaves in $CDCl_3$ S31

Spectrum S2. ^{1}H NMR spectrum of a mixture of *N*-*trans*-feruloyltyramine (HCAA1) and *N*-*trans*-3-methoxytyramine (HCAA2) isolated from *N. benthamiana* leaves in CD_3OD S32

Spectrum S3. ^{1}H and ^{13}C NMR spectra of *N*-*trans*-feruloyltyramine (HCAA1) in acetone-d_6 .... S33

Spectrum S4. ^{1}H and ^{13}C NMR spectra of *N*-*trans*-3-methoxytyramine (HCAA2) in acetone-d_6 .... S34

Supplementary References **S35**

Supplementary Materials and Methods

General

^{1}H NMR spectra were recorded at room temperature on an Inova 500 (500 MHz) or an Inova 600 (600 MHz) spectrometer. Chemical shifts are reported in ppm downfield from tetramethylsilane using solvent resonance as internal standards ($CDCl_3$: δ 7.26 ppm, CD3OD: δ 3.31 ppm, or acetone-d_6: δ 2.05 ppm).
Proton-decoupled ^{13}C NMR spectra were recorded at room temperature on an Inova 500 (126 MHz) spectrometer. Chemical shifts are reported in ppm downfield from tetramethylsilane using solvent resonance as internal standards (acetone-d_6: δ 29.84 ppm).
All synthetic, work-up and purification procedures were carried out with at least reagent-grade solvents. Standard column chromatography techniques with SilicaFlash® P60 silica gel (40-63 μm) were used for purification unless otherwise noted.

Materials

PyBOP (Acros Organics), tyramine (Aldrich), ferulic acid (Mfg Spectrum Chemical), 3-methoxytyramine (Combi-Blocks) were used as received without further purification. EA and its biosynthetic intermediates were used as prepared previously.[1] $CDCl_3$, CD_3OD and acetone-d_6 were purchased from Cambridge Isotope Laboratories.

Agro-infiltrated *N. benthamiana* RNA isolation, RNA-seq dataset preparation, and cDNA synthesis

N. benthamiana leaves were infiltrated with *Agrobacterium* strains harboring pEAQ-HT (empty vector), pEAQ-HT:GFP, and all EA pathway genes (pEAQ-HT-GG:DIR-PLR-SDH, pEAQ-HT-GG:CYP719A23-OMT3, pEAQ-HT:CYP71CU1, pEAQ-HT:OMT1, pEAQ-HT:2-ODD, pEAQ-HT:CYP71BE54, and pEAQ-HT:CYP82D61).[1] (For 5-day-post-infiltration harvest samples, 1 mM coniferyl alcohol [substrate to the EA pathway] was infiltrated on 4 dpi, the day before harvest.) The infiltrated leaves were harvested on 2 or 5 days post-agro-infiltration. WT (uninfiltrated) leaves were harvested on 0 or 2 days post-infiltration (with respect to the infiltrated leaves). Biological triplicates for each sample (total of 24 samples) consisted of leaves from different plants. Agro-infiltrated or WT *N. benthamiana* leaves were flash frozen in liquid nitrogen and ground with a micropestle in 2-ml Eppendorf tubes. Total RNA was extracted using the Spectrum Plant Total RNA kit (Sigma Aldrich) according to the manufacturer's instructions. The extracted RNA samples were analyzed using an RNA 6000 Nano chip on a 2100 Bioanalyzer (Agilent) for quality assessment.

RNA-seq libraries for each sample were prepared from the total RNA using the NEBNext Ultra Directional RNA Library Prep Kit for Illumina. The resulting library samples were analyzed using a DNA 1000 chip on a 2100 Bioanalyzer for quality checking, and the pooled library was analyzed using a High Sensitivity DNA chip to assess quality and quantity. Next-generation sequencing (paired-end, 2x150 bp) was performed using a single lane on the Illumina HiSeq4000 system at the Stanford Functional Genomics Facility. The resulting raw read data were deposited in the

National Center for Biotechnology Information (NCBI) Sequence Read Archive (SRA) database under the BioProject accession PRJNA863901.

cDNA was prepared from the extracted total RNA pool from all samples using the SuperScript IV First-Strand Synthesis system (Invitrogen) according to the manufacturer's instructions.

RNA-seq data analysis

The quality of the raw HiSeq4000 reads were assessed with FastQC.[2] The raw reads were trimmed using trimmomatic[3] with the following parameters:

```
ILLUMINACLIP:$TRIMMOMATICROOT/adapters/TruSeq3-PE.fa:2:30:10 LEADING:5
TRAILING:5 SLIDINGWINDOW:4:5 MINLEN:50 HEADCROP:14
```

The trimmed reads were assessed for quality by FastQC and mapped to a reference dataset composed of the full genomic CDS list of *N. benthamiana*[4] and CDS regions of the pEAQ-HT vectors (EV, GFP, all 10 EA genes from *P. hexandrum*[1]) using bowtie2[5] with the final alignment rate of 60%. (Note: the gene-level alignment rate was much lower than the expected had the reads been mapped to a reference transcriptome (transcript-level). However, we decided to take advantage of the fully annotated list of coding sequences for our purpose of biosynthetic pathway identification and engineering, not discovery of new genes.) The mapped reads were quantified using kallisto,[6] and the resulting effective counts were normalized using the TMM method in edgeR[7] using a Perl script "`abundance_estmates_to_matrix.pl`" provided in the Trinity package.[8,9] The resulting abundance estimates matrix (with TMM-normalized, $\log_2$-transformed [effective counts+1], 21 samples x 42,851 CDSs) was used as an input for clustering and co-expression analysis.

N. benthamiana gene primer design and qRT-PCR

For CA pathway genes, the *P. hexandrum* CA pathway genes[10] were used to identify *N. benthamiana* homologs by BLAST (tblastx, >80% identity) in the reference CDS list.[4] Whenever possible, primers were designed to encompass multiple allelic variants of the target gene to capture the general expression level change at the gene level, not at the allele level (see Table S3).

For primer design, primer-BLAST[11] with the following parameters was used: PCR product size, 80-150 bp; primer melting temperatures (Tm), min 54 °C, opt 57 °C, max 60 °C; database, nr; organism, Nicotiana benthamiana (taxid:4100).

N. benthamiana leaves were flash frozen in liquid nitrogen and stored at -80°C until RNA extraction. The total RNA was extracted using the Spectrum Plant Total RNA kit (Sigma Aldrich) with random hexamers, and cDNA templates were prepared using the SuperScript IV First-Strand Synthesis system (Invitrogen). Total RNA amounts were quantified by NanoDrop 2000c (Thermo Fisher) with absorbance values measured at 280 nm in TE buffer at pH 8.0. For quantitative RT-PCR, each sample was supplied at 10 ng of total cDNA (as estimated by total RNA quantity) per well with gene-specific primers for reference housekeeping genes[12] (PP2A, L23, and F-Box), Actin, CA pathway genes, and UGT genes (see Table S3 for primers) using SensiMix SYBR Hi-Rox Kit (Bioline) with Tm = 60 °C on QuantStudio3 Real-Time PCR Systems (Applied Biosystems). According to the methods developed by Liu and Shi et al.,[12] the geometric mean of

the expression levels of the three reference genes was used to normalize the expression values of each gene per sample, and relative expression values were determined with respect to the average expression values of the control triplicates (GFP) using the comparative Ct method ($2^{-\Delta\Delta Ct}$).[13]

Cloning pET28a constructs for *Sr*UGT71BE1 and *Nb*UGT expression in *E. coli*

The gene fragment containing the open reading frame of *Sr*UGT71E1 (AY345976.1) and overlaps with pET28a was purchased from Twist Bioscience. Eleven homologs of *Sr*UGT71E1 in the *Nb* genome were identified by BLAST search (tblastx with the whole sequence or the functional domain [Pro240-Ile474, see below] of *Sr*UGT71E1 as query) against the *N. benthamiana* genomic coding sequence (CDS) list,[4] and their sequences are deposited in the NCBI GenBank database: UGT71AT3 (OP121095), UGT72B34 (OP121099), UGT93S1 (OP121102), UGT73A25 (OP121101), UGT73A24 (OP121100), UGT709Q1 (OP121104), UGT93T2 (OP121103), UGT71BA2 (OP121097), UGT72AX1 (OP121098), UGT71AT4 (OP121096), UGT71AT2 (OP121094). The genes were amplified from the *N. benthamiana* cDNA templates with the stop codon included for un-tagged expression (see Table S1 for primers used). The *Sr*UGT71E1 gene fragment and the *Nb*UGT amplicons were inserted into pET28a (kanamycin^R) pre-digested with NdeI and XhoI or pre-linearized by PCR amplification to remove the *C*-terminal 6xHis-Tag. The resulting constructs were transformed into *E. coli* 5-alpha competent cells, and plasmid DNAs were isolated for sequence verification and transformation into *E. coli* BL21 (DE3) competent cells (New England BioLabs).

>Functional domain of *Sr*UGT71E1 (AY345976.1)
CCGATTTTGAACCTTGAAAACAAAAAAGACGATGCTAAAACCGACGAGATTATGAG
GTGGTTAAATGAGCAACCGGAAAGCTCGGTTGTGTTTTTATGTTTCGGAAGCATGGG
TAGCTTTAACGAGAAACAAGTGAAGGAGATTGCGGTTGCGATTGAAAGAAGTGGAC
ATAGATTTTTATGGTCGCTTCGTCGTCCGACACCGAAAGAAAAGATAGAGTTTCCGA
AAGAATATGAAAACTTGGAAGAAGTTCTTCCAGAGGGATTCCTTAAACGTACATCA
AGCATCGGGAAGGTGATCGGGTGGGCCCCACAAATGGCGGTGTTGTCTCACCCGTC
AGTTGGTGGGTTTGTGTCGCATTGTGGTTGGAACTCGACATTGGAGAGTATGTGGTG
TGGGGTTCCGATGGCAGCTTGGCCATTATATGCTGAACAAACGTTGAATGCTTTTCT
ACTTGTGGTGGAACTGGGATTGGCGGCGGAGATTAGGATGGATTATCGGACGGATA
CGAAAGCGGGGTATGACGGTGGGATGGAGGTGACGGTGGAGGAGATTGAAGATGG
AATTAGGAAGTTGATGAGTGATGGTGAGATTAGAAATAAGGTGAAAGATGTGAAAG
AGAAGAGTAGAGCTGCGGTTGTTGAAGGTGGATCTTCTTACGCATCCATTGGAAAAT
TCATCGAGCATGTATCGAATGTTACGATT

E. coli lysate screening assays for *Nb*UGT candidates

Recombinant UGTs were prepared as described previously.[14] Briefly, a single colony per *E. coli* BL21 (DE3) cells harboring each of the pET28a:*Nb*UGT constructs was inoculated into 6 ml (x2) of LB medium containing 50 µg/ml kanamycin and grown at 30 °C with shaking at 300 rpm until OD_{600} = 0.6, at which point the culture was induced with 1 mM isopropyl β-D-1-thiogalactopyranoside (IPTG) and further incubated for 3 h. The cultures were cooled in ice water and pelleted at 3000 rpm at 4 °C for 10 min. The pelleted cells were resuspended in 880 µl of resuspension buffer (0.5 mM potassium phosphate buffer at pH 7.2 containing 0.2% v/v Triton X-

100 [Sigma] and 100 µg/ml lysozyme [Thermo Fisher]) and incubated for 20 min. The cultures were sonicated 3 x 10 sec on ice. The crude cell lysates were further concentrated by ultracentrifugation (Amicon Ultra-0.5mL 10 kD), flash frozen in liquid nitrogen and stored at -80 °C until use.

The cell lysates were used directly in an activity assay using (–)-matairesinol as the substrate. The whole cell lysate was added to the standard glucosyltransferase assay buffer (50 mM potassium phosphate buffer at pH 7.2 containing 3 mM $MgCl_2$ and 10 µg/ml BSA) at 5-fold dilution along with 50 µM (–)-matairesinol. The reactions were initiated by addition of UDP-glucose (Sigma) to a final concentration of 1 mM and incubated at 30 °C for 2 h with shaking at 300 rpm. The reactions were quenched with an equal volume of acetonitrile and filtered with 0.45 µm Nylon filters for LC-MS analysis.

LC-MS data analysis

For targeted analysis, LC-MS data were analyzed using MassHunter Qualitative Analysis software (Agilent). For untargeted metabolomic analysis, the data files were exported into the mzData file format on the MassHunter Qualitative Analysis software and processed in R with the XCMS[15,16] (Scripps Center for Metabolomics) and CAMERA[17] packages with the following sample codes:

```
xset <- xcmsSet(filelist, #filelist contains a list of
                          #path/to/datafile.mzData files
                method='centWave', ppm=40, snthresh=6, peakwidth=c(6,30))
sampnames(xset) <- rxnNames   # rxnNames is a list of sample name
                              # order of this list should match the order of
                              # filelist inputted for xcmsSet()
sampclass(xset) <- rxnClasses # rxnClasses is a list of sample groups
                              # (same group name is repeated for replicates)
                              # order of this list should match the order of
                              # rxnNames
xset.f <- xset %>% group(bw = 20, mzwid=0.01) %>%
            retcor(family = 's', plottype='m') %>%
            group(bw = 10) %>%
            fillPeaks() # require(dplyr) and require(tidyr)
diffreport  <- annotateDiffreport(xset.f, ppm=40, polarity="positive")
        # require(CAMERA)
reportList <- vector(mode = 'list', length = nrow(comp))
        # comp is a (number of comparisons) x 2 (class1, class2) data frame,
        # where each row contains two groups (classes) to be compared.
for (i in 1:nrow(comp)) {
    print(i)
    reportList[[i]] <- diffreport(xset.f, comp$class1[i], comp$class2[i],
sortpval=FALSE, metlin = TRUE) %>%
    mutate(Pair.ID = paste0(comp$class1[i],' vs ', comp$class2[i]),
               Pairwise_class1 = comp$class1[i],
               Pairwise_class2 = comp$class2[i])
}
#combines above data.frames into one large data.frame
reportDF <- bind_rows(reportList) %>% select(Pair.ID, Pairwise_class1,
Pairwise_class2, everything())
# diffreport is left joined to reportDF by "name" for pcgroup grouping
reportDF.annotated <- reportDF %>% left_join(annotatedDF.clean %>%
select(name,pcgroup),by=c("name"))
```

The resulting metabolomic matrix (log10-transformed abundances, 21 samples x 16,812 mass features) was used for further clustering analysis. The diffreports (`reportDF`) generated from pairwise comparison were joined with the annotated diffreport output (`diffreport`) from the CAMERA package for manual metabolite annotation.

Clustering analyses on metabolomic and transcriptomic data

All clustering analyses were performed in R with the default package. Principal component analysis was performed with the `prcomp()` function from expression data (TMM-normalized, log_2-transformed raw counts, centered) and metabolomic data (~16,000 mass features per sample typically identified by the XCMS package on a 20-min gradient method, log_2-transformed and pareto-scaled ion counts,[18,19] not centered). Hierarchical clustering was performed with the `hclust()` function from the aforementioned datasets, using Pearson's correlation as the distance metric and average-linkage method.

Preparative HPLC purification

For preparative HPLC purification, an Agilent 1260 Infinity preparative-scale HPLC system with an Agilent 1100 diode array detector and a Clipeus C18 10 µm 250 x 20 mm column (Higgins Analytical) was used. Unless otherwise noted, water with 0.1% formic acid (A) and acetonitrile with 0.1% formic acid (B) were used as mobile phase at a flow rate of 4.3 ml/min with the following method: 0-10 min, 20-30% B; 10-60 min, 30% B isocratic; 60-65 min, 30-97% B; and 65-80 min, 97% B.

For mass-triggered fraction collection, an Agilent single quad MSD 6120 was used with a split ratio of 200:1 and an isopump (Agilent 1260 Infinity II) with a flow rate of 0.3 ml/min and dilution factor of 15:1. MS parameters were as the following: peak width, 0.1 min; cycle time, 0.78 sec/cycle; drying gas, 350 °C and 12 L/min; nebulizer, 35 psig; capillary, 3000 V. For multisignal acquisition, concurrent SIM/scan modes were used. Signal 1 (positive ion SIM mode, 50% cycle time) on sample target masses with the following parameters: fragmentor, 70 V; gain 1. Signal 2 (positive ion scan mode, 50% cycle time) with the following parameters: mass range, 100-700 m/z; fragmentor, 70V; threshold, 150; step size 0.2; gain, 1; scan speed, 1300 u/sec.

Isolation of EA, HCAA1, and HCAA2 from *N. benthamiana* leaves

Three to four leaves per plant on twenty plants expressing *At*MYB85 and the EA pathway were harvested on 7 days post-infiltration. Leaf tissue was flash-frozen in liquid nitrogen and lyophilized for 3 days. Dry leaf tissue (5.88 g) was pulverized with mortar and pestle, and the ground material was combined with 250 mL of methanol and refluxed at 65 °C for one hour. After vacuum filtration, the methanolic extract was concentrated *in vacuo*. The crude residue was first purified by silica gel column chromatography (Biotage Selekt with Sfär silica HC Duo 20 µm 25 g) to fractionate EA from HCAA1 and HCAA2 with the following gradient: 0-3 column volumes (CV), 0-10% acetone/toluene; 3-23 CV, 10-40% acetone/toluene; 23-25 CV, 40-100% acetone/toluene; and 25-35 CV, 100% acetone. The fractions containing EA or HCAAs were each concentrated *in vacuo* and resuspended in minimal volume of methanol and acetonitrile for mass-

triggered prep HPLC purification to yield EA (1.0 mg) and an inseparable mixture of HCAA1 and HCAA2.

4'-demethyl epipodophyllotoxin, or etoposide aglycone (EA). ^{1}H NMR (600 MHz, $CDCl_3$) δ 6.87 (s, 1H), 6.55 (s, 1H), 6.29 (s, 2H), 6.00 (s, 1H), 5.97 (s, 1H), 5.01 (s, 1H), 4.87 (d, *J* = 3.4 Hz, 1H), 4.61 (d, *J* = 5.3 Hz, 1H), 4.41 – 4.30 (m, 2H), 3.77 (s, 6H), 3.27 (dd, *J* = 14.1, 5.3 Hz, 1H), 2.83 (dddd, *J* = 14.1, 11.0, 8.0, 3.4 Hz, 1H). HRMS (m/z): $[M+H]^+$ calcd. for $C_{21}H_{21}O_8^+$, 401.1231; found, 401.1237. All spectroscopic data were consistent with reported literature values.[1]

Synthetic procedures

N-trans-feruloyltyramine (HCAA1). Authentic standard of HCAA1 was prepared by following the protocols described by Pham et al.[20] Amide coupling of ferulic acid (500 mg, 2.57 mmol, 1 equiv.) and tyramine (353 mg, 2.57 mmol, 1 equiv.) afforded HCAA1 (519.7 mg, 65 %) as a white foam. TLC (acetone/toluene, 2:3 v/v): R_f = 0.40. ^{1}H NMR (500 MHz, Acetone-d_6) δ 8.35 (s, 1H), 8.12 (s, 1H), 7.47 (d, *J* = 15.6 Hz, 1H), 7.15 (d, *J* = 2.0 Hz, 1H), 7.08 – 7.02 (m, 3H), 6.84 (d, *J* = 8.1 Hz, 1H), 6.79 – 6.74 (m, 2H), 6.52 (d, *J* = 15.6 Hz, 1H), 3.86 (s, 3H), 3.54 – 3.46 (m, 2H), 2.75 (t, *J* = 7.4 Hz, 2H). ^{13}C NMR (126 MHz, Acetone-d_6) δ 166.6, 156.7, 149.1, 148.6, 140.5, 131.0, 130.5, 128.2, 122.6, 119.9, 116.09, 116.05, 111.2, 56.1, 42.0, 35.7. HRMS (m/z): $[M+H]^+$ calcd. for $C_{18}H_{20}NO_4^+$, 314.1387; found, 314.1394. All spectroscopic data were consistent with reported literature values.[20,21]

N-trans-feruloyl-3-methoxytyramine (HCAA2). Authentic standard of HCAA2 was prepared by following the protocols described by Pham et al.[20] Amide coupling of ferulic acid (500 mg, 2.57 mmol, 1 equiv.) and 3-methoxytyramine (523.4 mg, 2.57 mmol, 1 equiv.) afforded HCAA2 (786.6 mg, 89 %) as a yellow foam. TLC (acetone/toluene, 2:3 v/v): R_f = 0.45. ^{1}H NMR (500 MHz, Acetone-d_6) δ 8.30 (s, 1H), 7.59 (s, 1H), 7.49 (d, *J* = 15.7 Hz, 1H), 7.14 (d, *J* = 2.1 Hz, 1H), 7.04 (dd, *J* = 8.0, 2.1 Hz, 1H), 6.87 – 6.81 (m, 2H), 6.75 (d, *J* = 8.0 Hz, 1H), 6.67 (dd, *J* = 8.0, 2.1 Hz, 1H), 6.56 (d, *J* = 15.7 Hz, 1H), 3.84 (s, 3H), 3.79 (s, 3H), 3.53 (q, *J* = 7.1 Hz, 2H), 2.76 (d, *J* = 7.1 Hz, 2H). ^{13}C NMR (126 MHz, acetone) δ 166.7, 149.2, 148.6, 148.2, 145.9, 140.6, 131.6, 128.1, 122.6, 121.9, 119.8, 116.1, 115.7, 113.1, 111.2, 56.13, 56.11, 41.9, 36.1. HRMS (m/z): $[M+H]^+$ calcd. for $C_{19}H_{22}NO_5^+$, 344.1492; found, 344.1498. All spectroscopic data were consistent with reported literature values.[21]

Supplementary Discussion

Identification of *Nb*UGT73A24 activity on EA metabolites

In addition to EA, its biosynthetic intermediates bearing the 4'-hydroxy group (e.g. (–)-matairesinol, (–)-pluviatolide, and 4'-demethyl deoxypodophyllotoxin, see Figure S2A) we have produced heterologously in *N. benthamiana* leaves are metabolized to the glycosylated forms by putative UDP-glycosyltransferases (UGTs), as evidenced by similar metabolite profile patterns. One possibility for the increased yield with MYB85 is that product glycosylation is repressed by this TF. To test this hypothesis, we first sought to identify a *N. benthamiana* UGT that contributes to EA glycosylation. We considered that if the endogenous UGT enzyme was known, it could be targeted for mutation in plant lines modified for improved product yield. Previously, a UGT from *Stevia rebaudiana* (*Sr*UGT71E1) that had been reported to modify etoposide into etoposide-4'-O-glucoside[22] and we used this protein sequence as query for candidate proteins using a homology-based search. We identified and prioritized testing of 11 *N. benthamiana* homologs of *Sr*UGT71E1 whose expression increased upon *Agrobacterium tumefaciens* leaf infiltration (see Supplementary Materials and Methods). Of these, *Nb*UGT73A24 converted (–)-matairesinol into (–)-matairesinol-4'-O-glucose as measured by LC/MS analysis of leaf extracts (Figure S10). Furthermore, overexpression of *Nb*UGT73A24 with the EA pathway in *N. benthamiana* leaves resulted in increased levels of both EA-4'-O-glucoside and EA-4'-O-malonylglucoside. However, no apparent repression of *Nb*UGT73A24 was observed in leaves expressing *At*MYB85 and the EA pathway compared to the GFP control (Figure S11). These results suggest that transcriptional repression of *Nb*UGT73A24 by *At*MYB85 is likely not the regulatory mechanism that prevents EA glycosylation.

Supplementary Figures and Tables

Table S1. Primers used for pET28a constructs

Gene	Directionality	Sequence 5’-3’
NbUGT71AT3	Forward	ctggtgccgcgcggcagccat**ATG**AATGAACTAATTTTCATTCC
NbUGT72B34	Forward	ctggtgccgcgcggcagccat**ATG**GCGGAAACTGCTATAG
NbUGT93S1	Forward	ctggtgccgcgcggcagccat**ATG**GCTGAAAACTACATC
NbUGT73A25	Forward	ctggtgccgcgcggcagccat**ATG**ATGGCTCAGGGCCAC
NbUGT73A24	Forward	ctggtgccgcgcggcagccat**ATG**GGTCAGCTCCATATTTTC
NbUGT72AX1	Forward	ctggtgccgcgcggcagccat**ATG**GACATATCTACAACAACGGC
NbUGT71AT4	Forward	ctggtgccgcgcggcagccat**ATG**GACACAAAAAAAGCAGAGC
NbUGT71AT2	Forward	ctggtgccgcgcggcagccat**ATG**ACAACGAAAGCAGAGC
NbUGT709Q1	Forward	ctggtgccgcgcggcagccat**ATG**GACCATCCCTCTCCTC
NbUGT93T2	Forward	ctggtgccgcgcggcagccat**ATG**GATTGCAGCAAGTTGAAAC
NbUGT71BA2	Forward	ctggtgccgcgcggcagccat**ATG**AAAAAGGCAAAGGTAGTGTTC
NbUGT71AT3	Reverse	tcagtggtggtggtggtggtgctcgag**TTA**AGAAATATTTGAAATTATATCATTAATTAG
NbUGT72B34	Reverse	gatctcagtggtggtggtggtggtgctcgag**TCA**ATTGTATAACACCTTATTCTTC
NbUGT93S1	Reverse	gatctcagtggtggtggtggtggtgctcgag**TTA**TTGAGTAATGTGAGCAATAAAAG
NbUGT73A25	Reverse	tcagtggtggtggtggtggtgctcgag**TTA**ATGTCCAGTGGAACTATATGTACTTATATC
NbUGT73A24	Reverse	tcagtggtggtggtggtggtgctcgag**TTA**ATGATCAGTAGAACTATATGTACTTATATC
NbUGT72AX1	Reverse	gatctcagtggtggtggtggtggtgctcgag**TCA**ACCACACAATGACTGATC
NbUGT71AT4	Reverse	tttcgggctttgttagcagccggatcctcgag**CTA**CCTCTTTTTCCTCACTTCATTC
NbUGT71AT2	Reverse	ggctttgttagcagccggatcctcgag**TCA**AGAAATATTACTAATGACATCCTCAATTAG
NbUGT709Q1	Reverse	ctttgttagcagccggatcctcgag**TTA**TTCAATGCAATTAGATTTGATATCCTTCCCTC
NbUGT93T2	Reverse	tttcgggctttgttagcagccggatcctcgag**CTA**TCTTGTGATATGCGCGATG
NbUGT71BA2	Reverse	tttcgggctttgttagcagccggatcctcgag**TCA**AATCACAGCCAATAATTTATCAATC

Overlapping sequences for pET28a backbone are shown in lowercase letters; start and stop codons are bolded; and restriction sites are underlined.

Table S2. Primers used for pEAQ-HT constructs

Gene	Directionality	Sequence 5’-3’
*Sr*UGT71E1	Forward	attctgcccaaattcgcgaccggtATGTCCACCTCAGAGCTTG
*Sr*UGT71E1	Reverse	gaaaccagagttaaaggcctcgagTTAAATCGTAACATTCGATACATGCTC
*Nb*UGT73A24	Forward	attctgcccaaattcgcgaccggtATGGGTCAGCTCCATATTTTC
*Nb*UGT73A24	Reverse	gaaaccagagttaaaggcctcgagTTAATGATCAGTAGAACTATATGTACTTATATCTTC
*At*MYB58	Forward	tgcccaaattcgcgaccggtATGGGCAAAGGAAGAGCACCATGTTG
*At*MYB58	Reverse	ccagagttaaaggcctcgagTTAATGTATGAGGAGCTCGTAACTCTCCAAGAGTG
*At*MYB63	Forward	tgcccaaattcgcgaccggtATGGGGAAGGGAAGAGCACCTTGTTG
*At*MYB63	Reverse	ccagagttaaaggcctcgagTCAATGTATCATGAGCTCGTAGTTCTTCAAGAGTGATG
*At*MYB85	Forward	tgcccaaattcgcgaccggtATGGGGAGACAGCCATGCTGTGAC
*At*MYB85	Reverse	ccagagttaaaggcctcgagTCAAAACCCAAAATCATGAACACCAAAGTCTTGAC
*At*VND6	Forward	tgcccaaattcgcgaccggtATGGAAAGTCTCGCACACATTC
*At*VND6	Reverse	ccagagttaaaggcctcgagTTACGTGTGTGTATTTTGAGCCCAAG
*At*VND7	Forward	tgcccaaattcgcgaccggtATGGATAATATAATGCAATCGTCAATGCC
*At*VND7	Reverse	ccagagttaaaggcctcgagTTACGAGTCAGGGAAGCATCC
*At*MYB103	Forward	tgcccaaattcgcgaccggtATGGGTCATCACTCATGCTGCAAC
*At*MYB103	Reverse	ccagagttaaaggcctcgagTTAAAACGAAGAAGGGAAAGAAGAAGATAAGGCAG
*At*MYB46	Forward	tgcccaaattcgcgaccggtATGAGGAAGCCAGAGGTAGCCATTG
*At*MYB46	Reverse	ccagagttaaaggcctcgagTCATATGCTTTGTTTGAAGTTGAAGTAAAACGAAGGAAC
*Nb*MYB85a	Forward	attctgcccaaattcgcgaccggtATGGGGAGAAAACCTTCTTGTGACAAAGATG
*Nb*MYB85ab	Reverse	gaaaccagagttaaaggcctcgagTCAAACTTCCAACACCTTTGACCAATCTTGG
*Nb*MYB85b	Forward	attctgcccaaattcgcgaccggtATGGGGAGAAAACCTTGTTGTGACAAAGCTG
*Nb*MYB46a	Forward	attctgcccaaattcgcgaccggtATGAGGAAGCCAGATCATCC
*Nb*MYB46ab	Reverse	gaaaccagagttaaaggcctcgagTCATTCAACTGGAAAATCAAGGTAAGGTAAG
*Nb*MYB46b	Forward	attctgcccaaattcgcgaccggtATGAGGAAGCCAGTTCATCC

Overlapping sequences for pEAQ-HT backbone are shown in lowercase letters; start and stop codons are bolded; and restriction sites are underlined.

Table S3. Primers used for qPCR

Gene	Directionality	Sequence 5’-3’
*Nb*Actin	Forward	CCGGTGTCCTGAGGTCCTTT
*Nb*Actin	Reverse	CTCGTGGATTCCTGCAGCTT
*Nb*UGT71AT3	Forward	TGGGAAGTTTTGAGGCAGAG
*Nb*UGT71AT3	Reverse	TTCCTTTGGTGGAGATCTTCTT
*Nb*UGT72B34	Forward	AGTACCACTCATAGCTTGGC
*Nb*UGT72B34	Reverse	TTTCATTGGCTTTTGGCCTC
*Nb*UGT93S1	Forward	GCCGGTAAAGAAAGGGAAAC
*Nb*UGT93S1	Reverse	AAATTTCCAACTGTGGTGCC
*Nb*UGT73A25	Forward	GTT AAG GCC ACT ATT ATC ACA ACC C
*Nb*UGT73A25	Reverse	TAGCTGGGAATTTGATCAAACGG
*Nb*UGT73A24	Forward	GTTAAAGCCACTATAATCACAACGCC
*Nb*UGT73A24	Reverse	CAGCTGGGAATTTGATCAAACGAA
*Nb*UGT72AX1	Forward	GGATGCAATCCAATATGCCC
*Nb*UGT72AX1	Reverse	TGGTAATCTGCTGACGTGAA
*Nb*UGT71AT4	Forward	AACAGCTGCTGAACTCTTGT
*Nb*UGT71AT4	Reverse	ACTTGGAAATTTCGTCTTCGT
*Nb*UGT71AT2	Forward	ACGGTCGAAATAGCCAATCT
*Nb*UGT71AT2	Reverse	TCAGGTTCAAAGCTTCCCAT
*Nb*UGT709Q1	Forward	ATTCAAGCCGGAGAGATTCC
*Nb*UGT709Q1	Forward	AGAATGGAAAATCACGACGG
*Nb*UGT93T2	Reverse	TCATGACATCCCAACTCCTG
*Nb*UGT93T2	Forward	TCGCGCATATTCATACAAGC
*Nb*UGT71BA2	Forward	ATTGGTAAATGCTGCTGCTG
*Nb*UGT71BA2	Reverse	GTCCCCACAAATCTTGATGC
*Nb*PAL1-4	Forward	CAA GAG CTG GTG TTA AAG CTA G
*Nb*PAL1-4	Reverse	GTA GCA CCA AAA CCA GTA GTA AC
*Nb*C4H1-3	Forward	GCA GCT TTT CAA AGA TTA CTT TGT TG
*Nb*C4H1-3	Reverse	AGC CTC AAG AAT GTG ATC AAT G
*Nb*4CL1	Forward	TCTGGAGAGATTTGCATTCGAGG
*Nb*4CL2	Forward	GCCGGAGAAATCTGCATTAGAG
*Nb*4CL1-2	Reverse	CTGTGTAACCATCCTTCTTTGTCTATTG
*Nb*HCT1-2	Forward	GTCTTCACTTCATCAACACATGGTC
*Nb*HCT1	Reverse	AGTGGATCACGAGCATGGAG
*Nb*HCT2	Reverse	TTGAGGTGGATCACGAGCAC
*Nb*C3H	Forward	TGTTGACCCTCCAACAGAAA
*Nb*C3H	Reverse	CCATTGCCCATTCAACAGAG
*Nb*CCoA-OMT1	Forward	ATGGCTCTTCCTGATGATGG
*Nb*CCoA-OMT1	Reverse	TGTGAGCTAGTCCAGCTTTT
*Nb*CCoA-OMT2	Forward	TTGCTACTGCTCTTGCTCTT
*Nb*CCoA-OMT2	Reverse	TCAATTTTATGAGCCACGCC
*Nb*CCR1-2	Forward	GACGAGACTTGTTGGAGTGA
*Nb*CCR1-2	Reverse	ACTCCTTTCTCCCTTGCTTC
*Nb*CCR3	Forward	CACCTGTCACTGATGATCCA
*Nb*CCR3	Reverse	CACTACCCTTTGCACTTTGG
*Nb*CAD1	Forward	TGCTGTGGACTCTGTCATAC
*Nb*CAD1	Reverse	CCACAACTTCACCTACCACT
*Nb*CAD2	Forward	TATTGTGGACTCTGCCACTC
*Nb*CAD2	Reverse	CTTCTCCCACCACTTCATGT

For *N. benthamiana* CA pathway genes, numbering indicates all the labels of allelic variants the corresponding primers are encompassing.

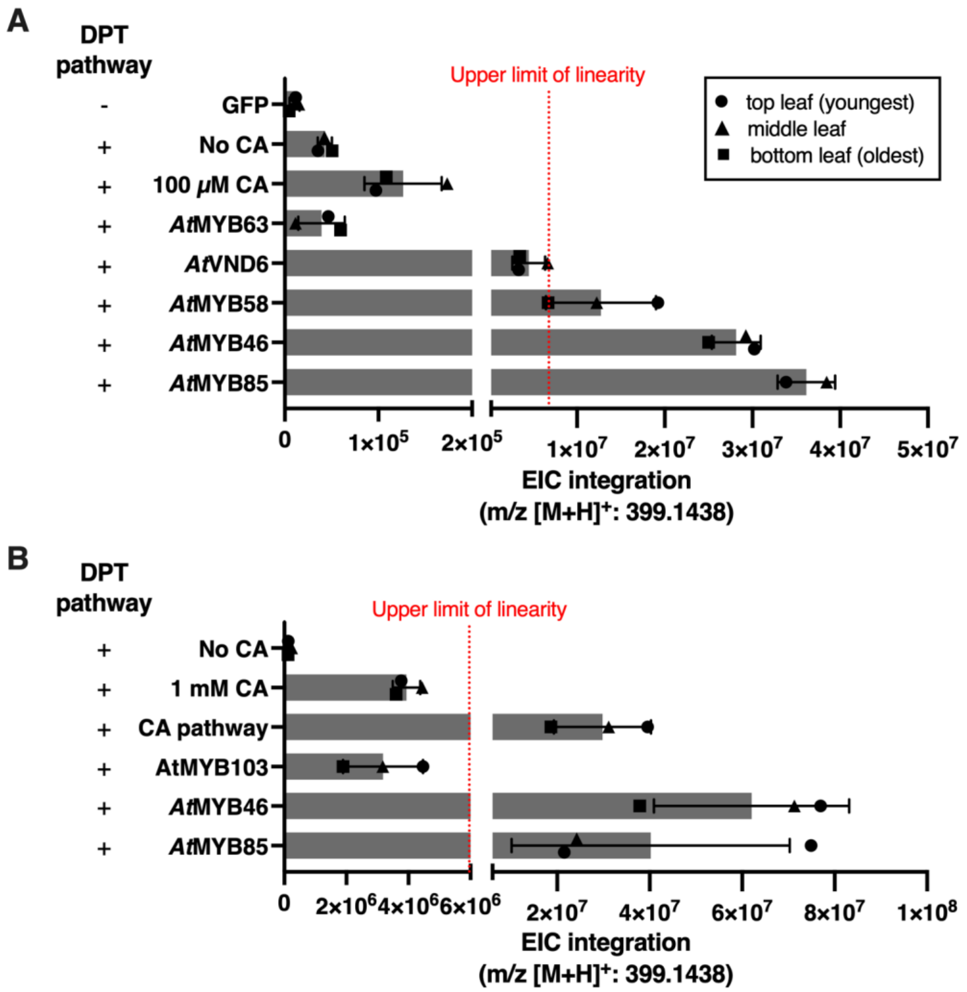

Figure S1. (–)-deoxypodophyllotoxin production with TF co-expression 5 days post-infiltration

(–)-deoxypodophyllotoxin (DPT) production is estimated based on LC-MS analysis of methanolic extracts from *N. benthamiana* expressing the DPT pathway (or none for GFP) along with exogenous coniferyl alcohol (CA) addition or co-expression of GFP, CA pathway genes or a transcription factor from *Arabidopsis*. DPT pathway: DPT biosynthetic genes (*Ph*DIR, *Ph*PLR, *Ph*SDH, *Ph*CYP719A23, *Ph*OMT3, *Ph*CYP71CU1, *Ph*OMT1, *Ph*2ODD); No CA: no exogenous addition of coniferyl alcohol (negative control).

(A) and (B) show data collected from two different trials. (Exception: the bottom leaf replicate of AtMYB85 co-expression sample in trial A was lost during sample preparation.) Bar heights indicate average of EIC peak areas of biological triplicates, and error bars standard deviations.

Note: Data points above ~$7x10^6$ are beyond the linear range of the standard curve for DPT as measured by LC-MS and reported here only for relative comparison to the controls. See Figure 2 for actual quantification calculated from LC-MS analysis of diluted samples.

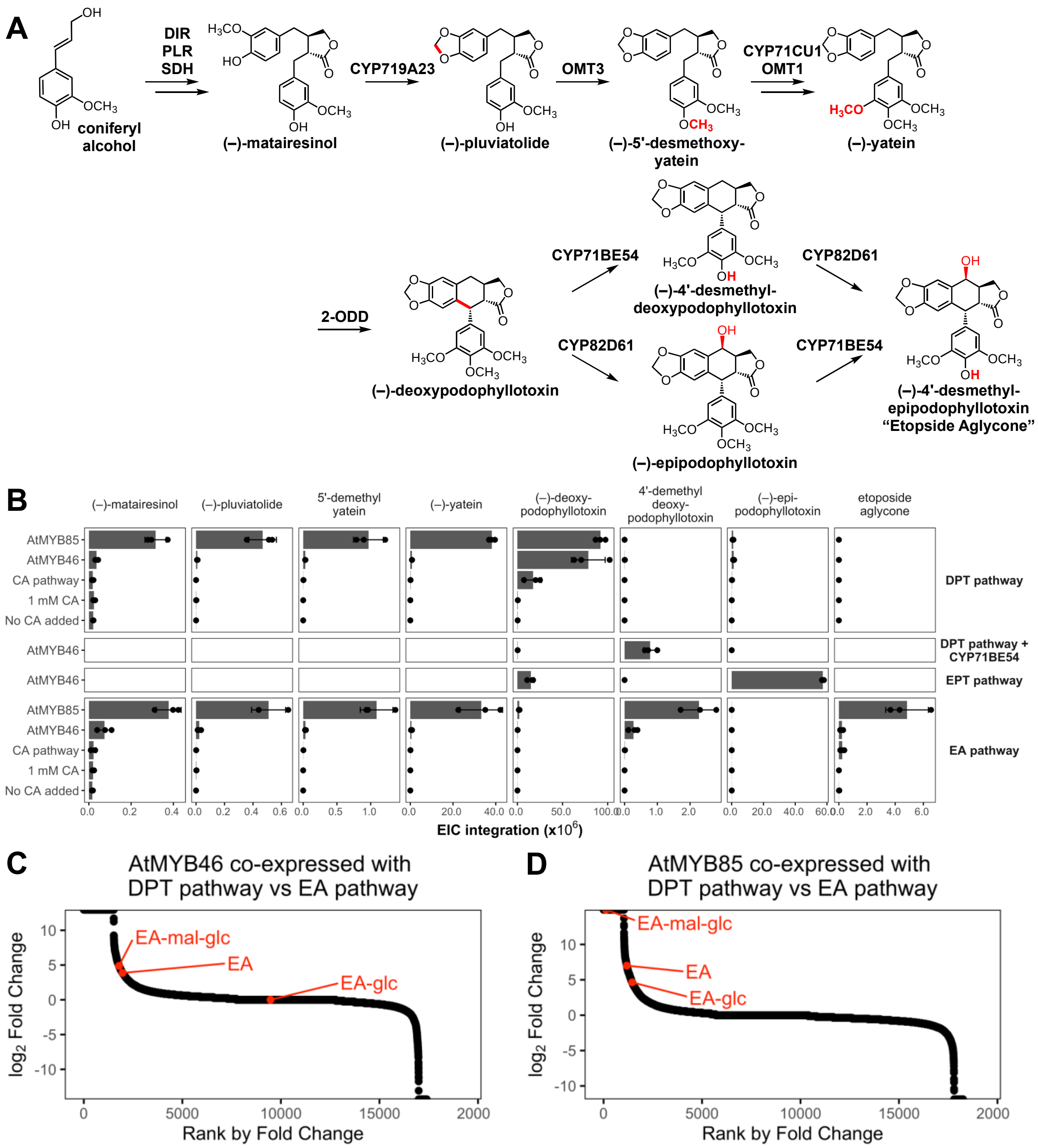

Figure S2. Accumulation of biosynthetic intermediates

(A) Full EA biosynthetic pathway with intermediate compound structures

(B) Biosynthetic intermediates in DPT or EA pathway accumulate when *At*MYB85 is co-expressed with the pathway. 4'-demethyl deoxypodophylltoxin and (–)-epipodophyllotoxin biosynthetic yields as promoted by *At*MYB46 co-expression with DPT pathway and CYP71BE54 or CYP82D61 are shown as reference for relative comparison of intermediate accumulation when the full EA pathway is expressed. Data points show EIC peak integration (m/z of [M+H]) as detected by LC-MS. Bar widths indicate mean of the biological triplicates, and error bars standard deviations. The y-axis shows the source or mechanism of substrate (coniferyl alcohol, CA)

availability increase. DPT pathway: DIR through 2-ODD; EPT pathway: DPT pathway and CYP82D61; and EA pathway: DPT pathway, CYP71BE54 and CYP82D61.

(C) Metabolite peaks found using untargeted metabolomics analysis that enriched in *N. benthamiana* leaf extracts expressing the EA pathway compared to the DPT pathway with *At*MYB46 co-expression. Mass features of EA-associated metabolites are highlighted in red.

(D) Metabolite peaks found using untargeted metabolomics analysis that enriched in *N. benthamiana* leaf extracts expressing the EA pathway compared to the DPT pathway with *At*MYB85 co-expression. Mass features of EA-associated metabolites are highlighted in red.

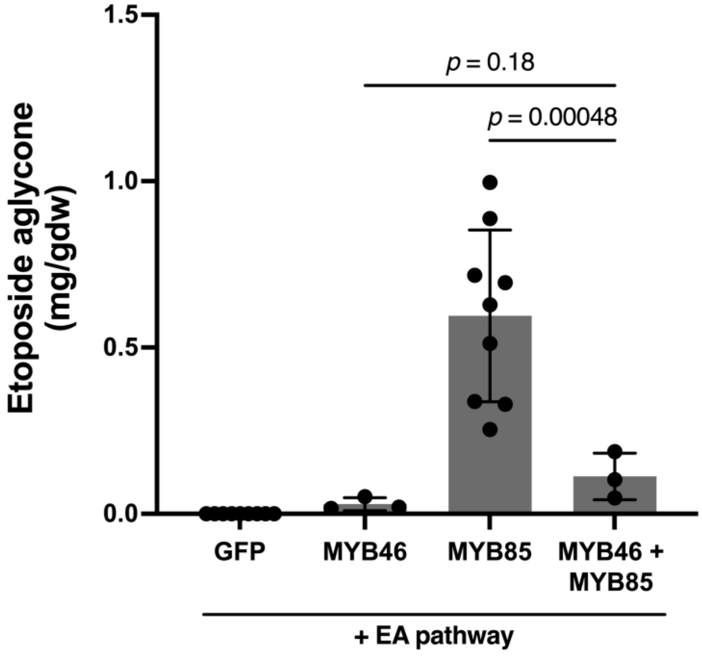

Figure S3. No synergistic effect between *At*MYB46 and *At*MYB85 on EA production
The x-axis shows gene or transcription factor co-expressed along with the EA pathway enzymes. Biosynthetic EA yields were quantified based on EIC peak integrations (m/z of $[M+H]^+$) as detected by LC-MS in comparison to standard curves. Bar heights indicate mean of biological triplicates (or n=9 for GFP and MYB85), and error bars standard deviations. Welch's t-test was performed assuming unequal variances, and the *p*-values are shown above each set of two samples compared.

A

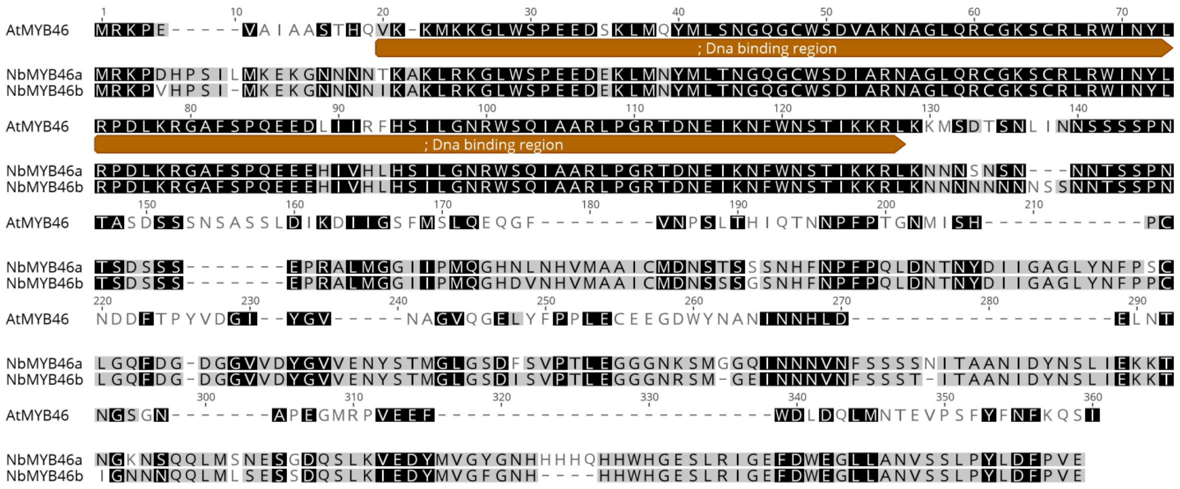

B

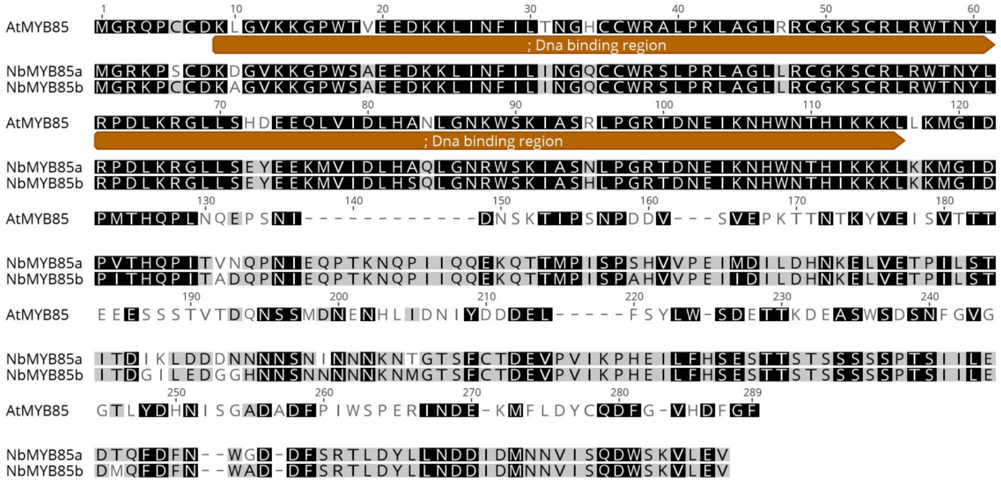

C

Homolog	% Identity		Reference
	Within motif	*overall*	
*Nb*MYB46a	87.2	39.0	*At*MYB46
*Nb*MYB46b	87.2	39.4	
*Nb*MYB85a	86.1	49.8	*At*MYB85
*Nb*MYB85b	85.2	49.8	

Figure S4. Protein sequence alignment of *N. benthamiana* homologs to *A. thaliana* transcription factors

(A) *At*MYB46 protein sequence alignment to *Nb*MYB46a and *Nb*MYB46b. DNA-binding domains of *At*MYB46 are highlighted as annotated per the ProRule database (annotation rule PRU00625).[23]

(B) *At*MYB85 protein sequence alignment to *Nb*MYB85a and *Nb*MYB85b. DNA-binding domains of *At*MYB85 are highlighted as annotated per the ProRule database (annotation rules PRU00625, PRU00133).[23]

(C) Percent identity at the amino acid level of *Nb* homologs to the respective *At* MYB proteins.

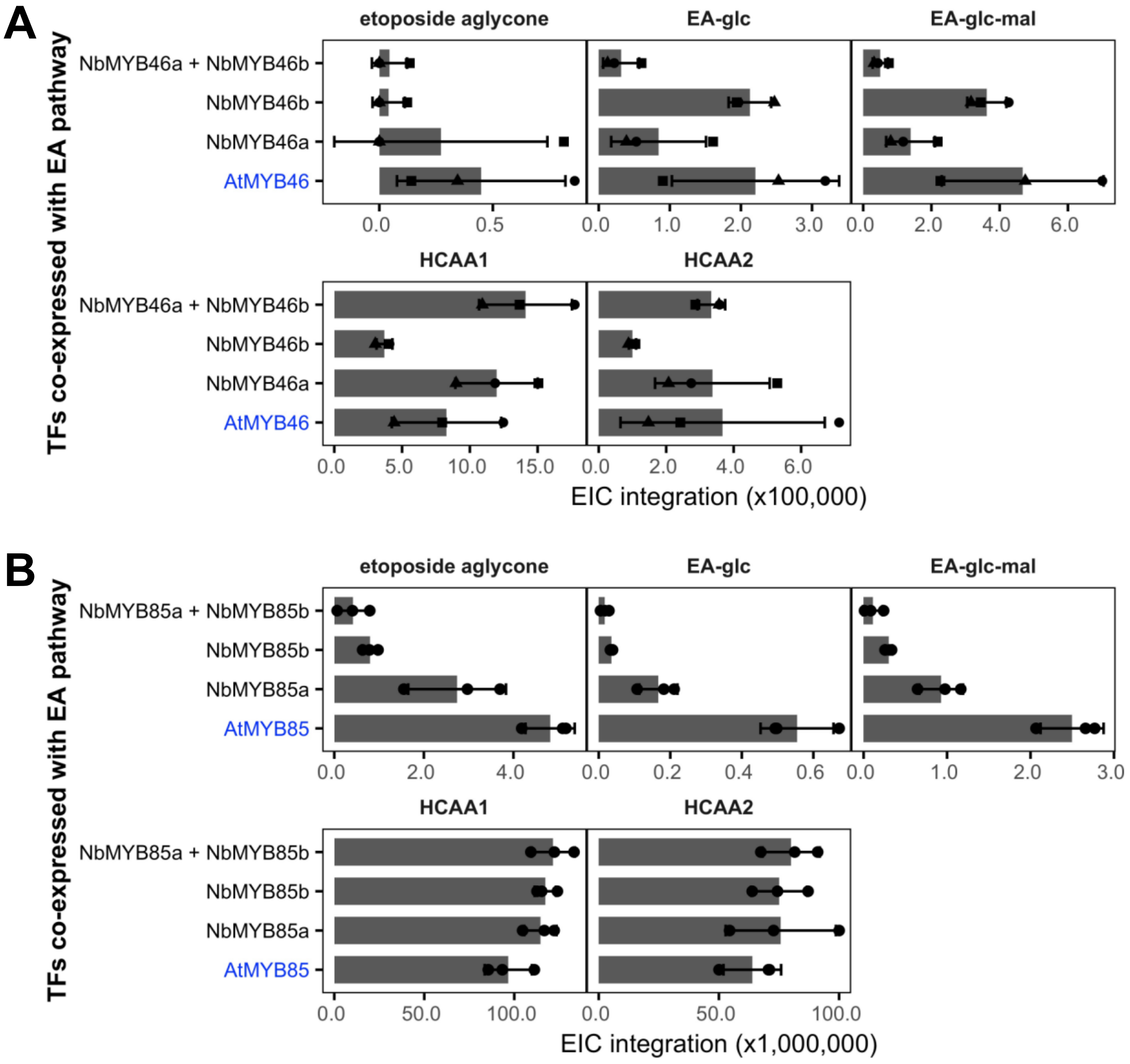

Figure S5. Biosynthesis of etoposide aglycone and side products promoted by *N. benthamiana* homologs of *At*MYB46 and *At*MYB85

(A) *N. benthamiana* MYB46 homologs of *At*MYB46. Relative metabolite concentrations are estimated by EIC peak integration (m/z of $[M+H]^+$ for aglycones or $[M+Na]^+$ for glycosides) as detected by LC-MS. Bar widths indicate mean of the biological triplicates, and error bars standard deviations.

(B) *N. benthamiana* MYB85 homologs of *At*MYB85. Relative metabolite concentrations are estimated by EIC peak integration (m/z of $[M+H]^+$ for aglycones or $[M+Na]^+$ for glycosides) as detected by LC-MS. Bar widths indicate mean of the biological triplicates, and error bars standard deviations.

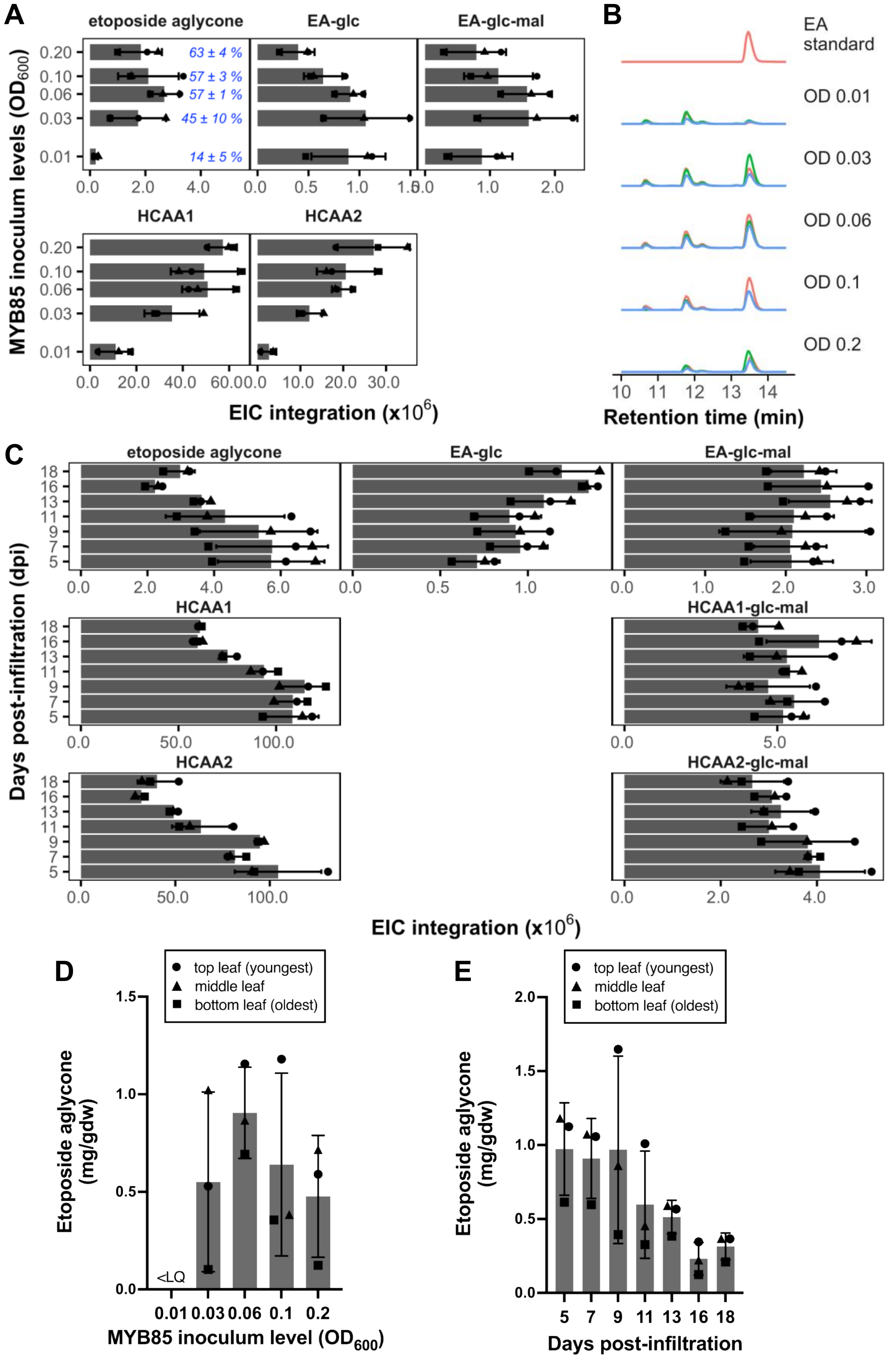
A
etoposide aglycone
EA-glc
EA-glc-mal
63 ± 4 %
57 ± 3 %
57 ± 1 %
45 ± 10 %
14 ± 5 %
HCAA1
HCAA2
MYB85 inoculum levels (OD600)
EIC integration (x10^6)
B
EA standard
OD 0.01
OD 0.03
OD 0.06
OD 0.1
OD 0.2
Retention time (min)
C
etoposide aglycone
EA-glc
EA-glc-mal
HCAA1
HCAA1-glc-mal
HCAA2
HCAA2-glc-mal
Days post-infiltration (dpi)
EIC integration (x10^6)
D
top leaf (youngest)
middle leaf
bottom leaf (oldest)
Etoposide aglycone (mg/gdw)
<LQ
MYB85 inoculum level (OD600)
E
top leaf (youngest)
middle leaf
bottom leaf (oldest)
Etoposide aglycone (mg/gdw)
Days post-infiltration

Figure S6. Optimization of EA biosynthetic yields

(A) EA and byproduct formation with increasing inoculum levels of the *Agrobacterium* strain harboring *At*MYB85 with respect to EA pathway strains. Data points show EIC peak integration for each corresponding ion species (m/z of $[M+H]^+$ for aglycones or $[M+Na]^+$ for glycosides) as detected by LC-MS. Bar widths indicate the mean of biological triplicates, and error bars standard deviations. Pseudo-purity (%) metric for EA is shown in blue as the mean of triplicates and standard deviations and estimated by the following formula: [EA]/([EA]+[EA-glc]+[EA-glc-mal]) where EIC peak integrations are used to estimate relative, not absolute, quantities.

(B) EICs of EA (m/z of $[M+H]^+$) with varying inoculum levels. Early eluting peaks at ~10.6 and ~11.8 min are in-source fragmentation of EA-glc and EA-glc-mal, respectively. All chromatograms are to scale, including the authentic standard at 75 µM.

(C) EA and byproduct formation over the time-course of 5-18 days post-infiltration. Data points show EIC peak integration for each corresponding ion species (m/z of $[M+H]^+$ for all but EA glycosides and $[M+Na]^+$ for EA glycosides) as detected by LC-MS. Bar widths indicate the mean of biological triplicates, and error bars standard deviations.

(D) Quantified EA yields with varying inoculum levels of *At*MYB85. Bar heights show the mean of biological triplicates, and error bars standard deviations. <LQ: below limit of quantification (< 0.04 mg/gdw).

(E) Quantified EA yields over the time-course of 5-18 days post-infiltration. Bar heights show the mean of biological triplicates, and error bars standard deviations.

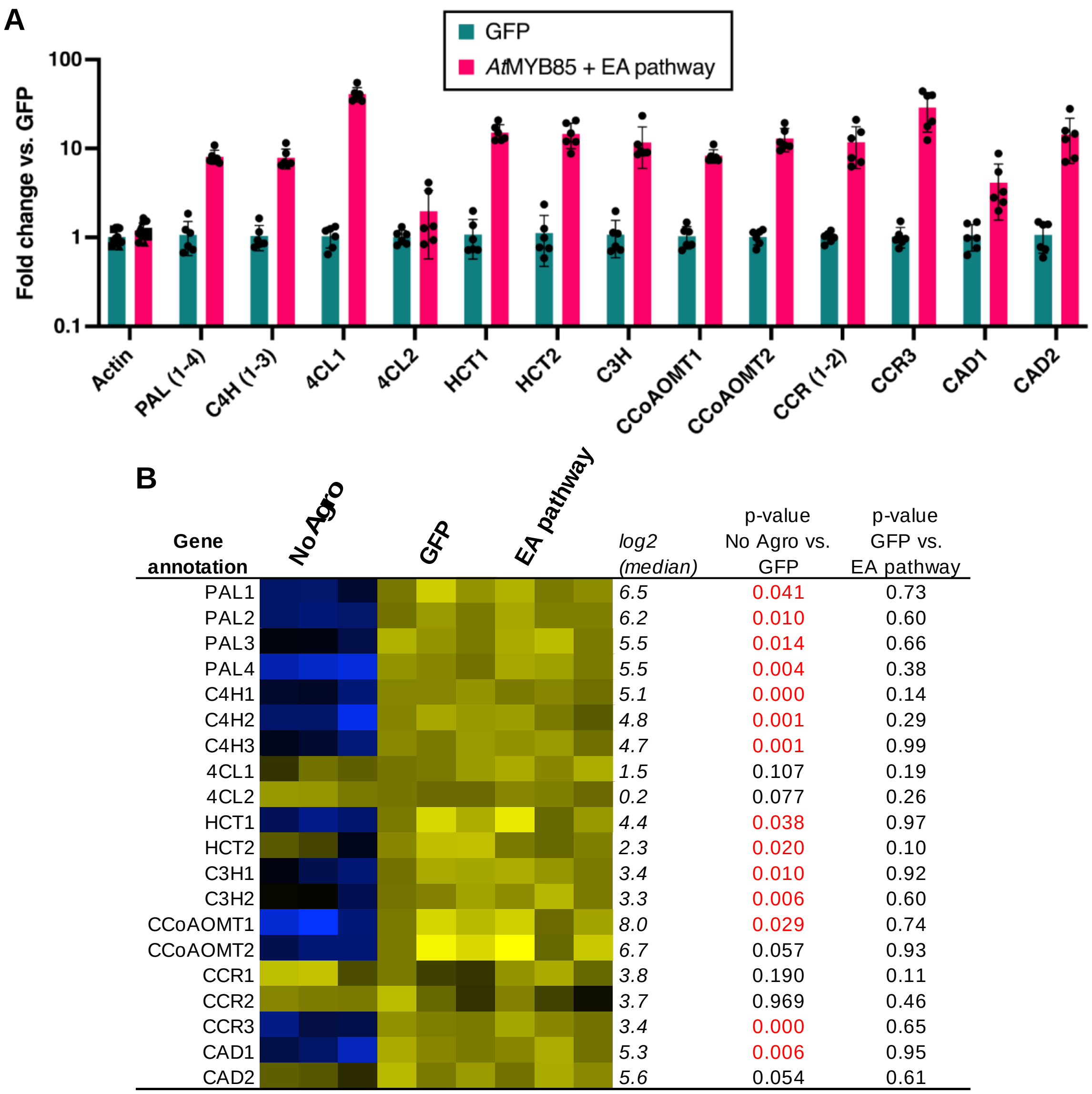

Gene annotation	log2 (median)	p-value No Agro vs. GFP	p-value GFP vs. EA pathway
PAL1	*6.5*	0.041	0.73
PAL2	*6.2*	0.010	0.60
PAL3	*5.5*	0.014	0.66
PAL4	*5.5*	0.004	0.38
C4H1	*5.1*	0.000	0.14
C4H2	*4.8*	0.001	0.29
C4H3	*4.7*	0.001	0.99
4CL1	*1.5*	0.107	0.19
4CL2	*0.2*	0.077	0.26
HCT1	*4.4*	0.038	0.97
HCT2	*2.3*	0.020	0.10
C3H1	*3.4*	0.010	0.92
C3H2	*3.3*	0.006	0.60
CCoAOMT1	*8.0*	0.029	0.74
CCoAOMT2	*6.7*	0.057	0.93
CCR1	*3.8*	0.190	0.11
CCR2	*3.7*	0.969	0.46
CCR3	*3.4*	0.000	0.65
CAD1	*5.3*	0.006	0.95
CAD2	*5.6*	0.054	0.61

Figure S7. Relative expression levels of CA pathway genes in *At*MYB85-expressing *N. benthamiana* leaves

(A) qRT-PCR gene expression analysis of eight canonical pathway enzymes for coniferyl alcohol (CA) biosynthesis in *N. benthamiana* leaves expressing *At*MYB85 and the EA pathway compared to those expressing GFP. Relative expression levels are normalized to the mean in the GFP control per gene. Bar heights indicate the mean of the biological replicates (n=6), and error bars standard deviations. For PAL, C4H, and CCR, primers were designed around high-similarity regions to amplify more than one allelic variant or paralog.

(B) Expression levels of the CA pathway genes in the *N. benthamiana* leaves harvested on 2 days post-infiltration (when infiltrated with *Agrobacterium*). The quantified effective counts were TMM-normalized, $\log_2$-transformed and median-centered for visualization. Two-tailed t-test assuming unequal variance was performed, and statistically significant differences are highlighted in red ($\alpha = 0.05$).

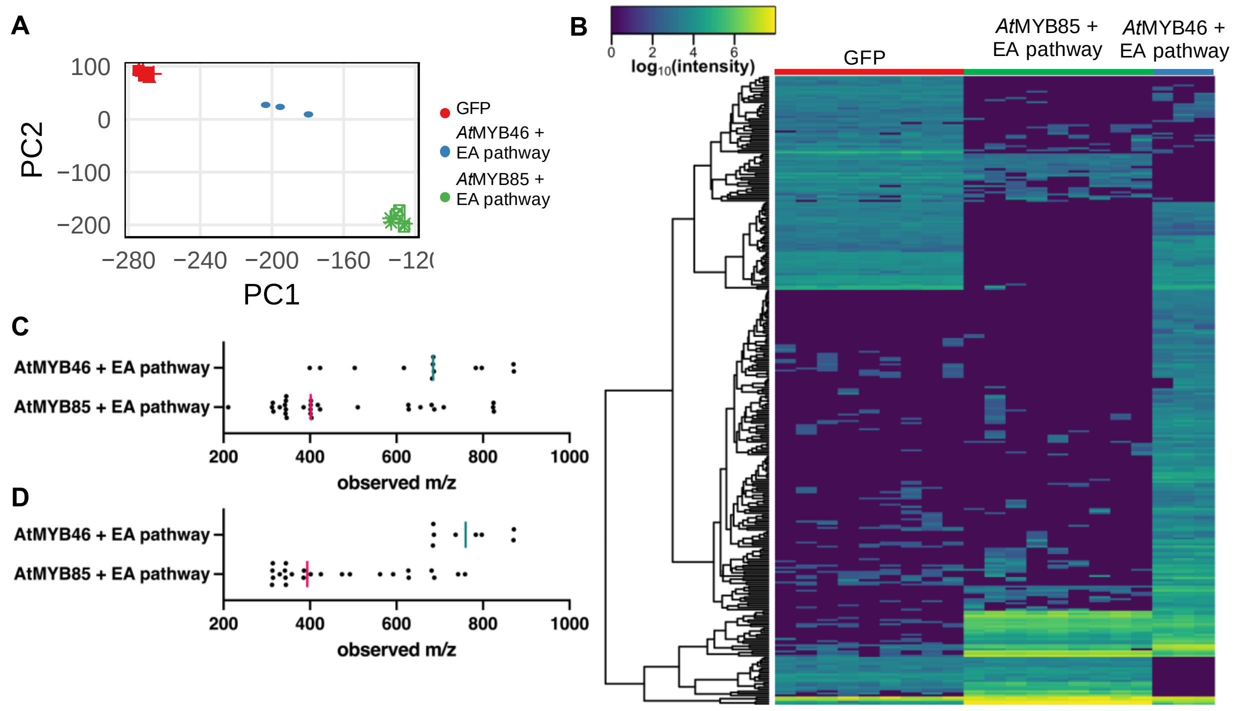

Figure S8. PCA and hierarchical clustering of *N. benthamiana* leaves based on metabolite composition

(A) Principal component analysis based on the composition of mass features in the plant extracts from leaves expressing GFP, *At*MYB85 and EA pathway, or *At*MYB46 and EA pathway.

(B) Hierarchical clustering analysis on the top 300 major loadings (mass features) in the PCA. GFP or *At*MYB85 + EA pathway: n=9, *At*MYB46 + EA pathway: n=3.

(C) Distribution of metabolite signals enriched in *At*MYB46 + EA pathway or *At*MYB85 + EA pathway samples with respect to the GFP control. Colored lined indicate the median. (see Table S4 and Table S5)

(D) Distribution of metabolite signals enriched in *At*MYB46 + EA pathway with respect to *At*MYB85 + EA pathway and vice versa. Colored lined indicate the median. (see Table S6 and Table S7)

Table S4. Metabolite peaks enriched in leaves expressing *At*MYB85 and EA pathway compared to those expressing GFP

GFP vs **MYB85-EA***

(avg intensity (MYB85-EA) > 1e6, p-value < 0.01, fold change > 200; top 50 out of 88 based on MYB85 intensity)

name	fold	p-value	m/z	R.T. (min)	avg intensity*	pcgroup	Annotation
M312T664	5684	1.5E-07	312.130	11.1	30255779.68	5	HCAA?
M330T664	1351	3.3E-07	330.134	11.1	10196187.65	5	HCAA?
M681T666	2752	9.4E-10	681.263	11.1	2831683.707	630	
M342T686	5876	2.8E-06	342.144	11.4	23580595.14	6	
M343T686	1463	3.4E-06	343.145	11.4	4995261.025	6	
M211T799	251	5.2E-03	211.100	13.3	3513753.568	1025	
M401T800	Inf	1.5E-04	401.137	13.3	4126730.39	18	EA [M+H]
M315T827	571	1.4E-05	315.152	13.8	13111960.8	2	
M628T828	736	5.5E-08	628.262	13.8	4619444.144	2	
M314T828	618	5.5E-05	314.148	13.8	62346302.67	2	HCAA1
M627T828	7914	3.9E-08	627.289	13.8	11350523.07	2	
M510T837	379	2.7E-05	510.228	14.0	2220546.795	40	
M344T845	3441	7.8E-08	344.160	14.1	71587838.57	1	HCAA2
M345T845	1562	1.1E-07	345.163	14.1	15553182.48	1	
M346T845	829	2.0E-07	346.164	14.1	2302315.419	1	
M344T846	10513	3.6E-05	344.325	14.1	2889937.409	1	
M709T846	923	1.4E-03	709.297	14.1	2832465.68	528	
M687T847	3029	3.2E-06	687.295	14.1	11240377.81	1	
M655T1121	732	2.5E-06	655.289	18.7	3484695.404	2989	M-glc-mal
M385T1140	798	4.7E-05	385.177	19.0	4766761.679	77	M
M401T1256_1	2415	3.8E-09	401.173	20.9	76985616.12	3	(–)-yatein [M+H]
M402T1256	1831	5.1E-09	402.177	20.9	19223130.32	3	
M418T1256	1307	5.7E-12	418.201	20.9	4030550.744	3	
M825T1256	30124	3.8E-09	825.327	20.9	3112806.156	3	
M823T1256	27184	2.4E-09	823.321	20.9	21624090.83	3	
M824T1256	51102	2.6E-09	824.323	20.9	10726081.4	3	
M403T1256	2733	8.0E-09	403.180	20.9	3369576.315	3	
M401T1256_2	178079	6.4E-06	401.353	20.9	2780736.67	3	
M423T1257	228	1.7E-05	423.158	21.0	9549292.093	3	

name: distinct mass feature label; fold: fold change between the two samples; R.T.: retention time; avg intensity: average mass peak intensity in the enriched sample (marked with an asterisk); pcgroup: grouping per CAMERA package.

Table S5. Metabolite peaks enriched in leaves expressing *At*MYB46 and EA pathway compared to those expressing GFP

GFP vs **MYB46-EA***

(avg intensity (MYB46-EA) > 1e6, p-value < 0.05, fold change > 4; 12 m/z peaks)

name	fold	p-value	m/z	R.T. (min)	avg intensity*	pcgroup	Annotation
M503T633	16	1.2E-02	503.20	10.6	1450975	25	
M682T642	37	2.1E-03	682.25	10.7	2009030	81	
M686T904	117	2.7E-02	686.26	15.1	1452714	3180	
M685T907	7027	2.5E-02	685.25	15.1	4163589	7	
M684T908	9458	2.4E-02	684.27	15.1	10649019	7	
M871T916	2662	1.5E-02	871.30	15.3	2452822	55	
M870T923	14681	2.2E-02	870.31	15.4	12385982	11	
M399T1068	55	3.3E-02	399.13	17.8	2329176	73	
M783T1182	15157	2.2E-02	783.26	19.7	1056366	190	
M617T1214	296	6.2E-03	617.22	20.2	1415859	173	
M423T1257	50	4.4E-02	423.16	21.0	2077334	3	(–)-yatein [M+Na]
M797T1300	2073	3.3E-02	797.28	21.7	1054366	1073	

name: distinct mass feature label; fold: fold change between the two samples; R.T.: retention time; avg intensity: average mass peak intensity in the enriched sample (marked with an asterisk); pcgroup: grouping per CAMERA package.

Table S6. Metabolite peaks enriched in leaves expressing AtMYB85 compared to AtMYB46 expressing leaves

MYB46 vs **MYB85***

(avg intensity > 1e6 (either sample group), p-value < 0.01, fold change > 200; 24 m/z peaks)

name	fold	p-value	m/z	R.T. (min)	avg intensity*	pcgroup	Annotation
M474T509	419	1.2E-04	474.191	8.5	1723785	65	
M312T664	740	1.5E-07	312.130	11.1	30255780	5	[M-H2O+H]
M330T664	457	3.3E-07	330.134	11.1	10196188	5	[M+H]
M681T666	476	9.4E-10	681.263	11.1	2831684	630	[2M+Na]
M313T684	204	2.3E-07	313.131	11.4	6103671	702	
M342T686	667	2.9E-06	342.144	11.4	23580595	6	
M343T686	230	3.5E-06	343.145	11.4	4995261	6	
M562T758	208	9.8E-07	562.208	12.6	4698396	26	HCAA1-glc-mal
M314T760	380	6.3E-04	314.148	12.7	1776001	26	
M592T767	208	6.2E-06	592.220	12.8	4153705	20	HCAA2-glc-mal
M344T776	664	1.7E-03	344.160	12.9	1577394	53	
M492T792	272	4.2E-07	492.218	13.2	4973455	27	
M628T828	711	5.5E-08	628.262	13.8	4619444	2	
M314T829	Inf	1.5E-03	314.308	13.8	1484687	2	HCAA1
M627T828	699	4.0E-08	627.289	13.8	11350523	2	
M344T846	1719	3.6E-05	344.325	14.1	2889937	1	HCAA2
M687T847	1093	3.2E-06	687.295	14.1	11240378	1	
M758T913	396	2.9E-04	758.271	15.2	1074388	101	
M358T938	517	1.5E-03	358.157	15.6	3181977	1300	
M742T1030	210	9.6E-06	742.276	17.2	2450778	64	
M386T1138	484	1.5E-05	386.182	19.0	1180705	77	
M385T1140	387	4.7E-05	385.177	19.0	4766762	77	
M425T1141	340	8.9E-06	425.172	19.0	1406818	77	
M401T1256_2	Inf	6.4E-06	401.353	20.9	2780737	3	(–)-yatein associated

name: distinct mass feature label; fold: fold change between the two samples; R.T.: retention time; avg intensity: average mass peak intensity in the enriched sample (marked with an asterisk); pcgroup: grouping per CAMERA package.

Table S7. Metabolite peaks enriched in leaves expressing *At*MYB46 compared to *At*MYB85 expressing leaves

MYB46* vs MYB85

(avg intensity > 1e6 (either sample group), p-value < 0.05, fold change > 5; 8 m/z peaks)

name	fold	p-value	m/z	R.T. (min)	avg intensity*	pcgroup	Annotation
M686T904	14	2.9E-02	686.261	15.1	1452714	3180	[M+3]
M685T907	39	2.7E-02	685.248	15.1	4163589	7	[M+2]
M684T908	24	2.6E-02	684.266	15.1	10649019	7	[M+1]
M871T916	14	1.7E-02	871.304	15.3	2452822	55	
M870T923	31	2.4E-02	870.308	15.4	12385982	11	
M783T1182	86	2.3E-02	783.264	19.7	1056366	190	
M797T1300	242	3.4E-02	797.280	21.7	1054366	1073	
M737T1490_1	5	1.7E-08	736.509	24.8	1643901	5127	

name: distinct mass feature label; fold: fold change between the two samples; R.T.: retention time; avg intensity: average mass peak intensity in the enriched sample (marked with an asterisk); pcgroup: grouping per CAMERA package.

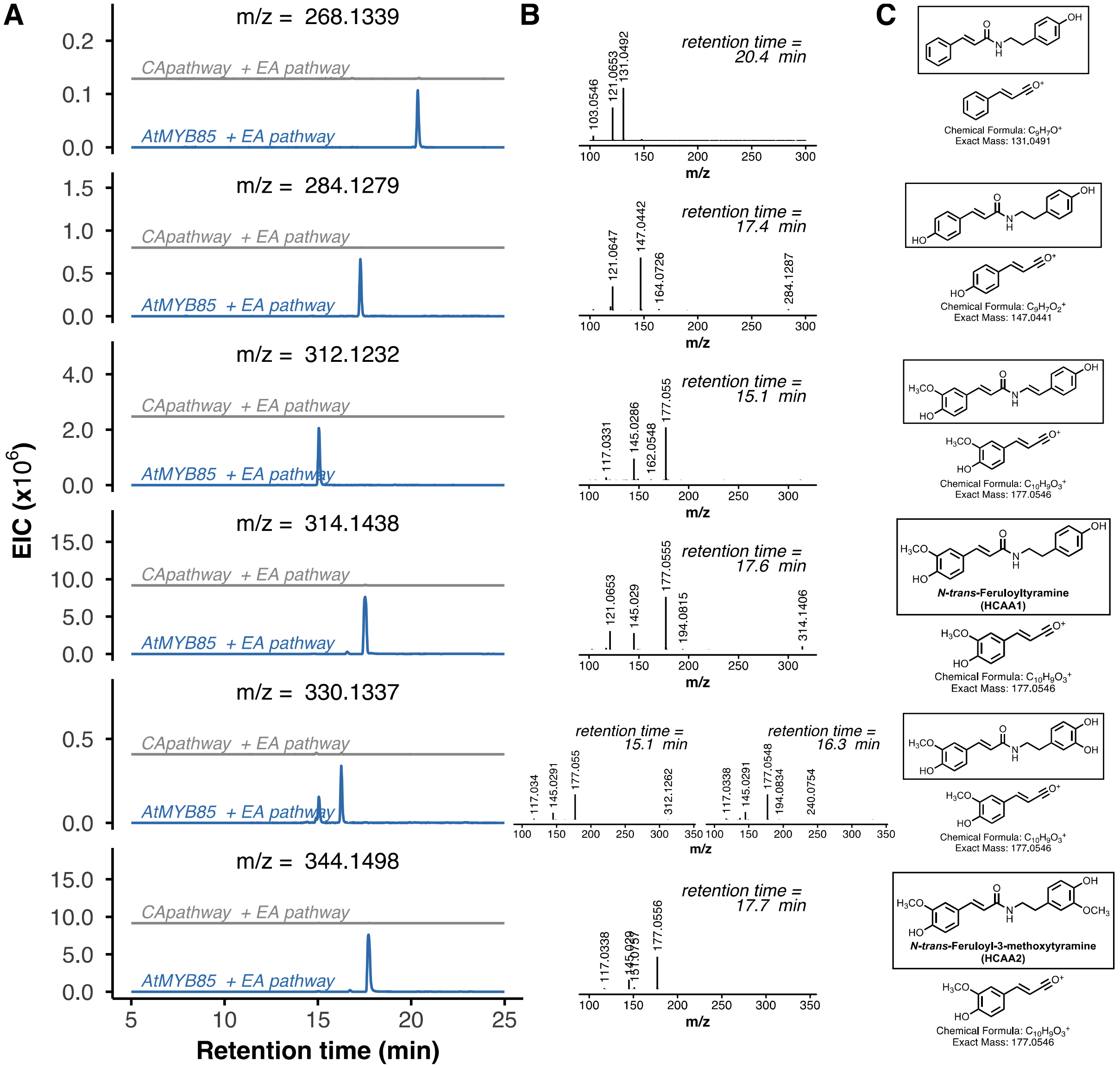

Figure S9. MS/MS fragmentation analysis of HCAA-like metabolites enriched in *At*MYB85-expressing samples

(A) EICs of the HCAA-like metabolites (m/z of the corresponding $[M+H]^+$ ion species shown) enriched in *N. benthamiana* leaves expressing *At*MYB85 and EA pathway compared to CA pathway overexpression.

(B) MS/MS spectra of the major EIC peaks shown in (A) at 20 V.

(C) Putative structure assignment based on the fragmentation patterns shown in boxes for each metabolite (per row), with the putative structures for the fragment ions shown below.

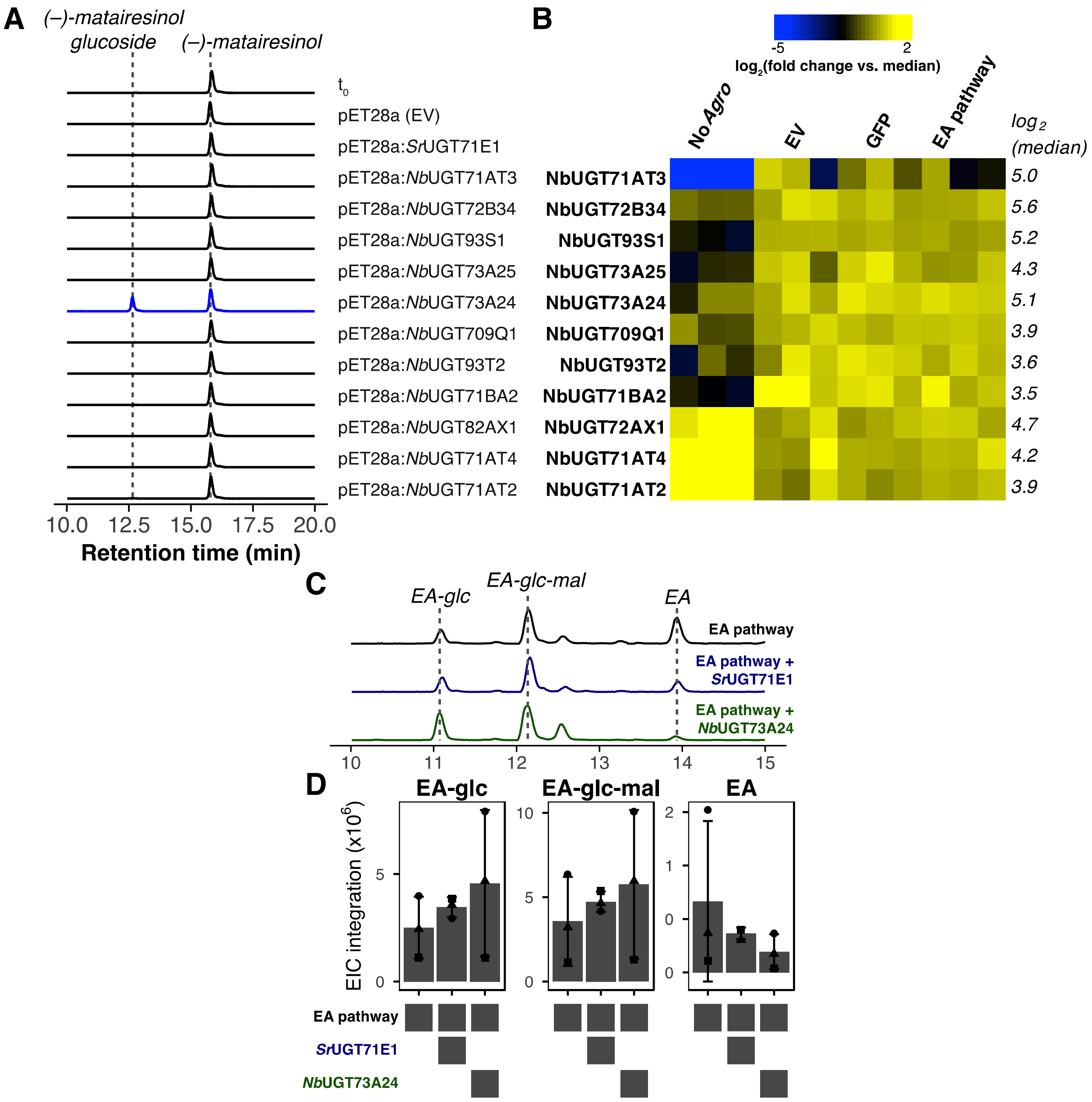

Figure S10. Identification of *Nb*UGT73A24 responsible for host glycosylation activity

(A) EICs of (–)-matairesinol (m/z $[M+H]^+$: 359.1489) from UGT activity assay with *E. coli* lysate expressing empty vector (EV) or *Nb*UGT candidate after 0 (top) or 90 min incubation. In-source fragmentation of (–)-matairesinol glucoside indicates presence of the glucoside product. The chromatogram traces are normalized per reaction.

(B) Expression levels of the candidate *Nb*UGTs in the *N. benthamiana* leaves harvested on 2 days post-infiltration (when infiltrated with *Agrobacterium*). The leaf tissue samples were either not infiltrated (no *Agro*) or infiltrated with *Agrobacterium* strains harboring empty pEAQ-HT vector (EV), GFP, or EA pathway genes. The quantified effective counts were TMM-normalized, $\log_2$-transformed and median-centered for visualization.

(C) EICs of etoposide aglycone (m/z $[M+H]^+$: 401.1231) from plant extracts collected from the *N. benthamiana* leaves expressing EA pathway only, EA pathway with *Sr*UGT71E1, and EA pathway with *Nb*UGT73A24. EICs are normalized per sample.

(D) Data points correspond to EIC integration at corresponding m/z values for EA-glc (etoposide aglycone 4'-glucoside, $[M\text{-glc}+Na]^+$: 585.1579), EA-glc-mal (etoposide aglycone 4'-malanoyl glucoside, $[M\text{-glc-mal}+Na]^+$: 671.1583), and etoposide aglycone ($[M+H]^+$: 401.1231). Bar heights indicate the mean, and error bars standard deviations of the biological triplicates.

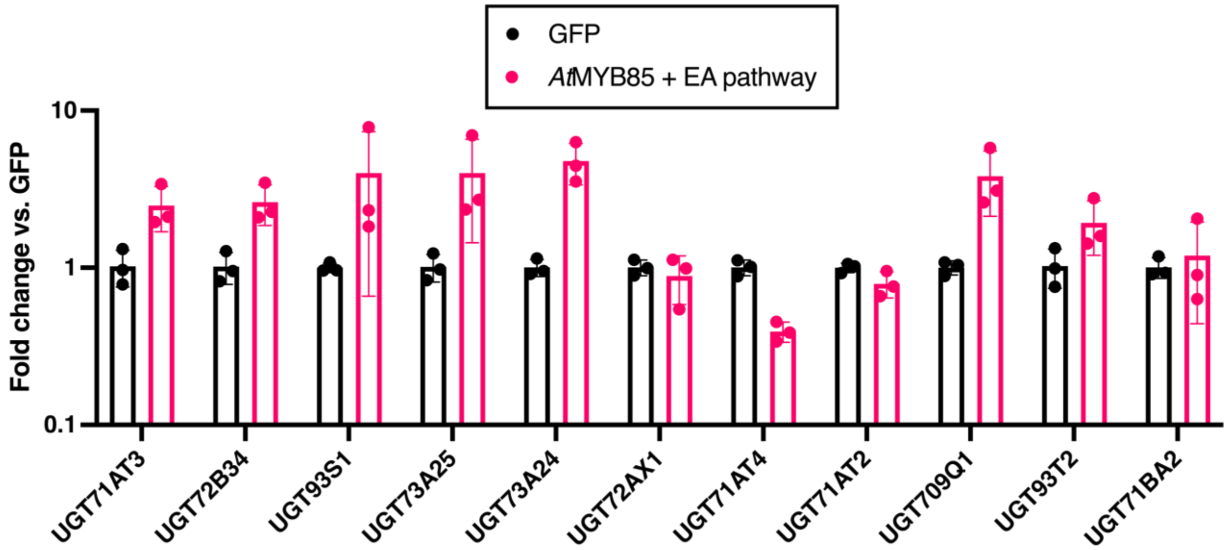

Figure S11. Relative expression levels of UGT homologs in *At*MYB85-expressing *N. benthamiana* leaves

qRT-PCR gene expression analysis of *Nb*UGT homologs of *Sr*UGT71BE1 in *N. benthamiana* leaves expressing *At*MYB85 and the EA pathway compared to those expressing GFP. Relative expression levels are normalized to the mean of triplicates in the GFP control per gene. Bar heights indicate the mean of the biological triplicates, and error bars standard deviations.

Supplementary Spectra

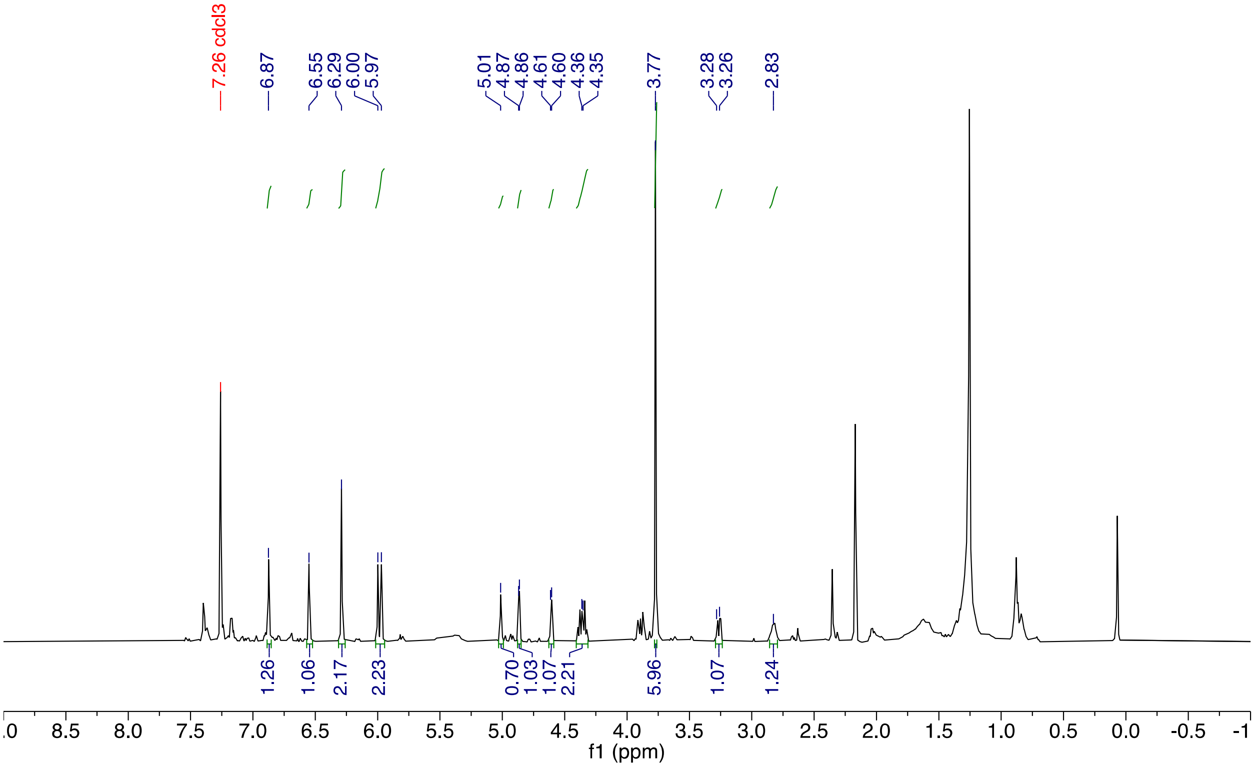

Spectrum S1. ^{1}H NMR spectrum of isolated etoposide aglycone from *N. benthamiana* leaves in $CDCl_3$

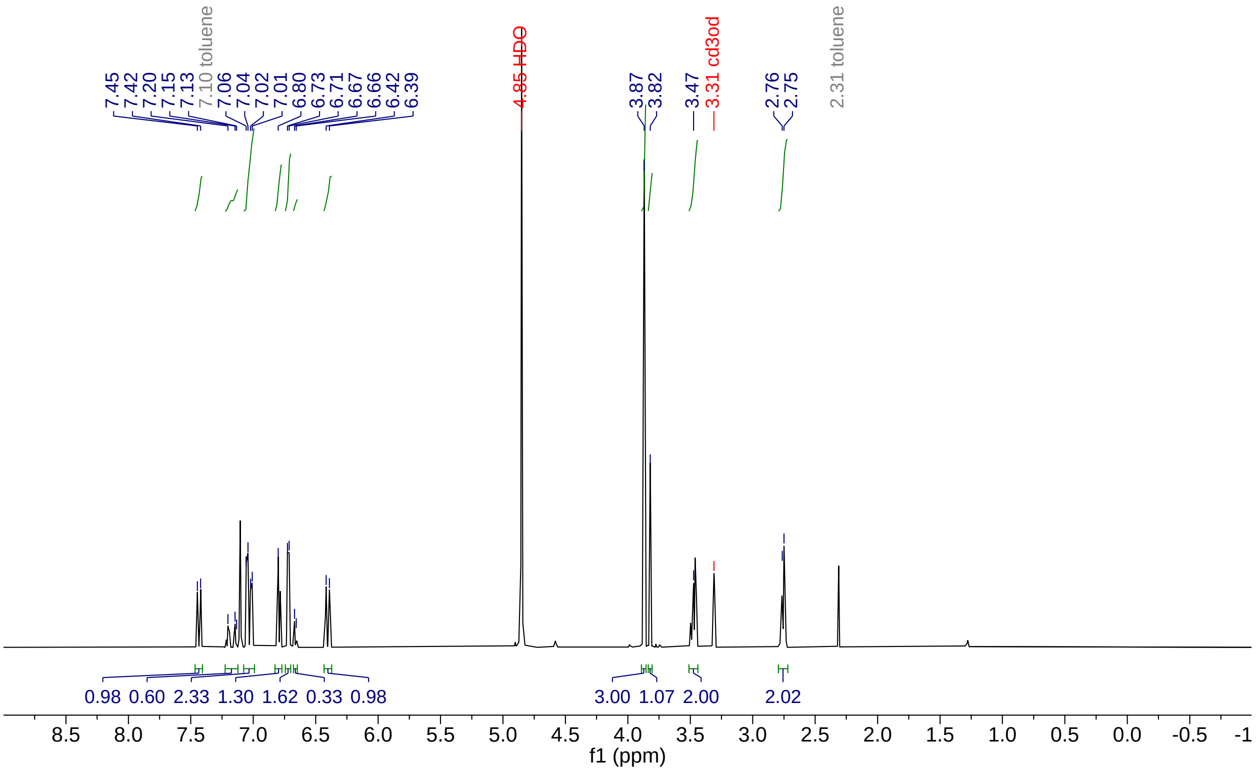

Spectrum S2. ^{1}H NMR spectrum of a mixture of *N-trans*-feruloyltyramine (HCAA1) and *N-trans*-3-methoxytyramine (HCAA2) isolated from *N. benthamiana* leaves in CD_3OD

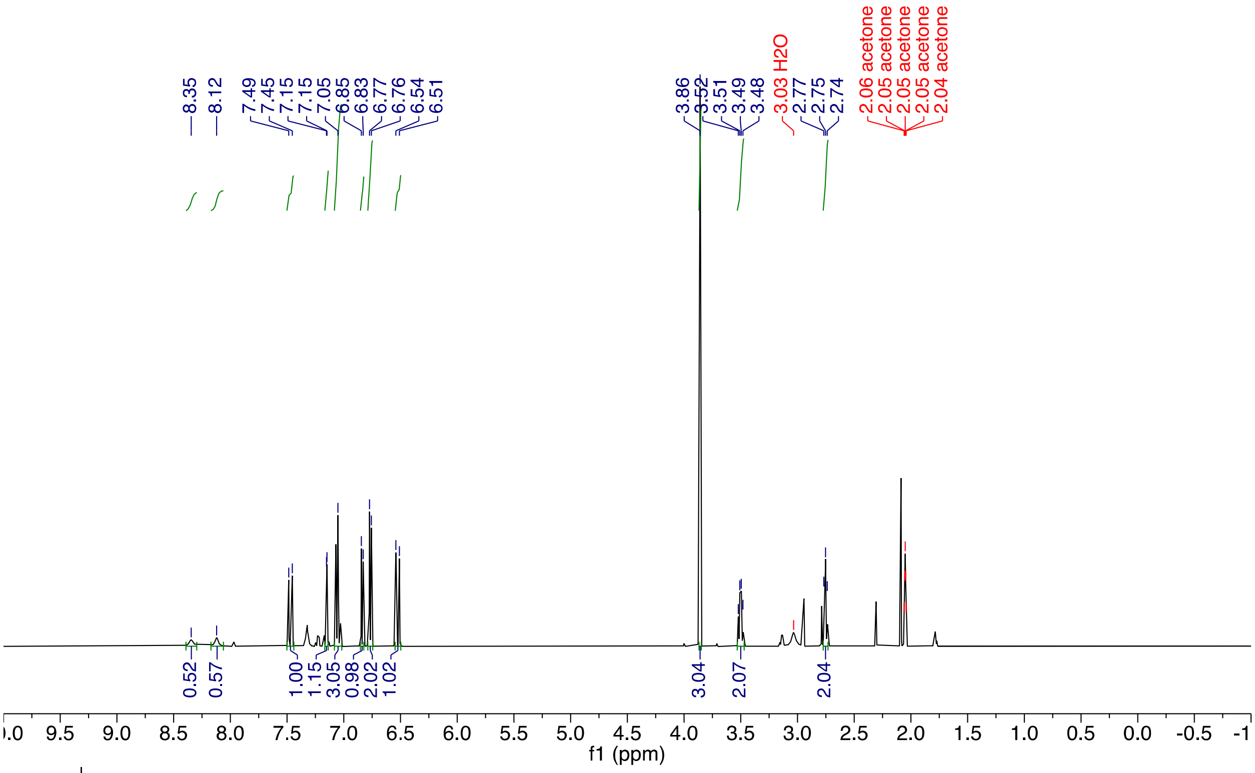

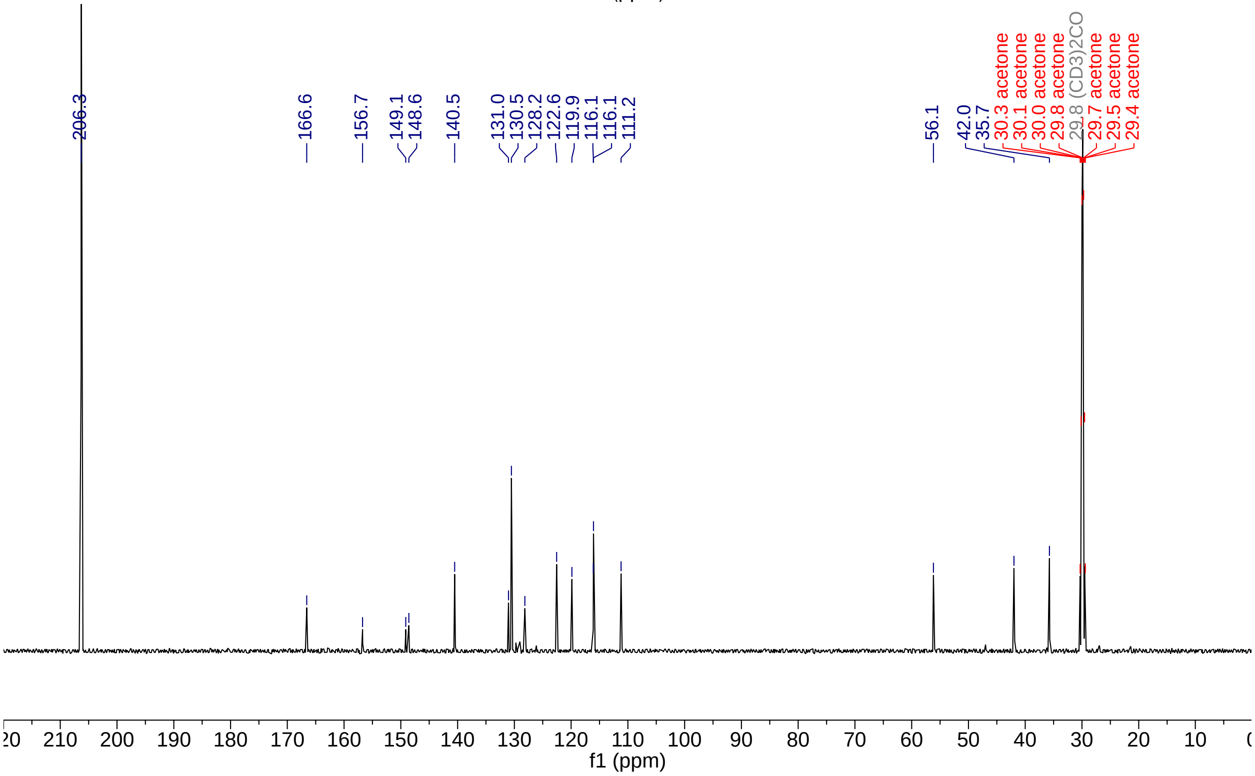

Spectrum S3. ^{1}H and ^{13}C NMR spectra of *N-trans*-feruloyltyramine (HCAA1) in acetone-d_6

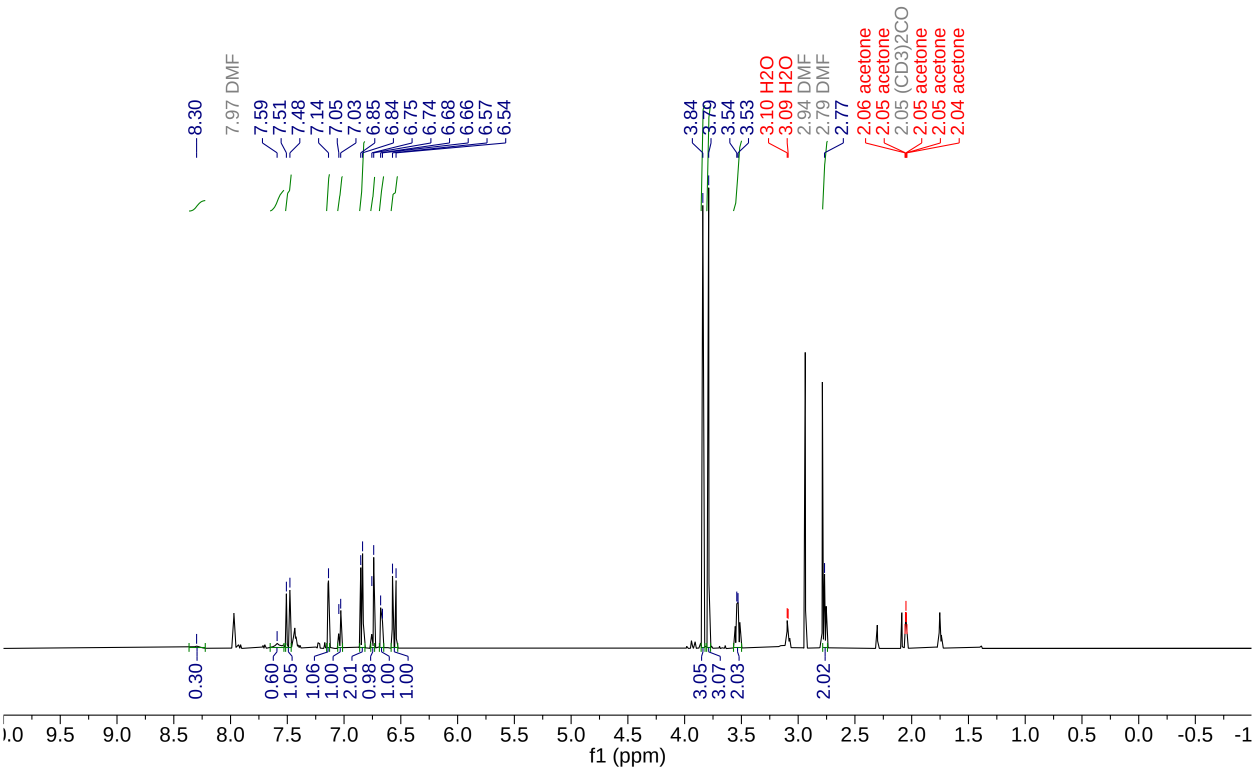

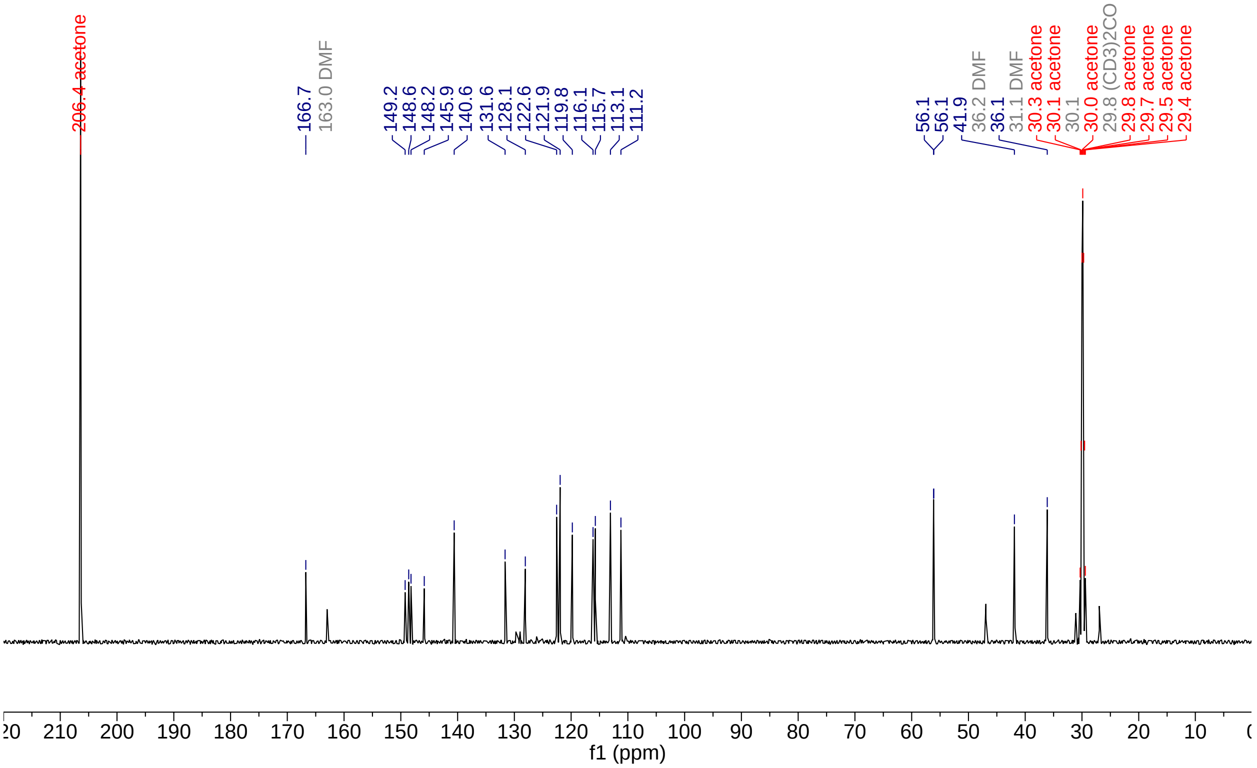

Spectrum S4. ^{1}H and ^{13}C NMR spectra of *N-trans*-3-methoxytyramine (HCAA2) in acetone-d_6

Supplementary References

(1) Lau, W.; Sattely, E. S. Six Enzymes from Mayapple That Complete the Biosynthetic Pathway to the Etoposide Aglycone. *Science* **2015**, *349* (6253), 1224–1228. https://doi.org/10.1126/science.aac7202.

(2) Andrews, S. *FastQC: A Quality Control Tool for High Throughput Sequence Data [Online]*. http://www.bioinformatics.babraham.ac.uk/projects/fastqc/.

(3) Bolger, A. M.; Lohse, M.; Usadel, B. Trimmomatic: A Flexible Trimmer for Illumina Sequence Data. *Bioinformatics* **2014**, *30* (15), 2114–2120. https://doi.org/10.1093/bioinformatics/btu170.

(4) Kourelis, J.; Kaschani, F.; Grosse-Holz, F. M.; Homma, F.; Kaiser, M.; Hoorn, R. A. L. van der. Homology-Guided Re-Annotation Improves the Gene Models of the Alloploid Nicotiana Benthamiana. *bioRxiv* **2019**, 373506. https://doi.org/10.1101/373506.

(5) Langmead, B.; Salzberg, S. L. Fast Gapped-Read Alignment with Bowtie 2. *Nat Methods* **2012**, *9* (4), 357–359. https://doi.org/10.1038/nmeth.1923.

(6) Bray, N. L.; Pimentel, H.; Melsted, P.; Pachter, L. Near-Optimal Probabilistic RNA-Seq Quantification. *Nat Biotechnol* **2016**, *34* (5), 525–527. https://doi.org/10.1038/nbt.3519.

(7) Robinson, M. D.; McCarthy, D. J.; Smyth, G. K. EdgeR: A Bioconductor Package for Differential Expression Analysis of Digital Gene Expression Data. *Bioinformatics* **2010**, *26* (1), 139–140. https://doi.org/10.1093/bioinformatics/btp616.

(8) Grabherr, M. G.; Haas, B. J.; Yassour, M.; Levin, J. Z.; Thompson, D. A.; Amit, I.; Adiconis, X.; Fan, L.; Raychowdhury, R.; Zeng, Q.; Chen, Z.; Mauceli, E.; Hacohen, N.; Gnirke, A.; Rhind, N.; di Palma, F.; Birren, B. W.; Nusbaum, C.; Lindblad-Toh, K.; Friedman, N.; Regev, A. Full-Length Transcriptome Assembly from RNA-Seq Data without a Reference Genome. *Nat Biotechnol* **2011**, *29* (7), 644–652. https://doi.org/10.1038/nbt.1883.

(9) Haas, B. J.; Papanicolaou, A.; Yassour, M.; Grabherr, M.; Blood, P. D.; Bowden, J.; Couger, M. B.; Eccles, D.; Li, B.; Lieber, M.; MacManes, M. D.; Ott, M.; Orvis, J.; Pochet, N.; Strozzi, F.; Weeks, N.; Westerman, R.; William, T.; Dewey, C. N.; Henschel, R.; LeDuc, R. D.; Friedman, N.; Regev, A. De Novo Transcript Sequence Reconstruction from RNA-Seq Using the Trinity Platform for Reference Generation and Analysis. *Nat Protoc* **2013**, *8* (8), 1494–1512. https://doi.org/10.1038/nprot.2013.084.

(10) Schultz, B. J.; Kim, S.-Y.; Lau, W.; Sattely, E. S. Total Biosynthesis for Milligram-Scale Production of Etoposide Intermediates in a Plant Chassis. *J. Am. Chem. Soc.* **2019**, *141* (49), 19231–19235. https://doi.org/10.1021/jacs.9b10717.

(11) Ye, J.; Coulouris, G.; Zaretskaya, I.; Cutcutache, I.; Rozen, S.; Madden, T. L. Primer-BLAST: A Tool to Design Target-Specific Primers for Polymerase Chain Reaction. *BMC Bioinformatics* **2012**, *13* (1), 1–11. https://doi.org/10.1186/1471-2105-13-134.

(12) Liu, D.; Shi, L.; Han, C.; Yu, J.; Li, D.; Zhang, Y. Validation of Reference Genes for Gene Expression Studies in Virus-Infected Nicotiana Benthamiana Using Quantitative Real-Time PCR. *PLOS ONE* **2012**, *7* (9), e46451. https://doi.org/10.1371/journal.pone.0046451.

(13) Livak, K. J.; Schmittgen, T. D. Analysis of Relative Gene Expression Data Using Real-Time Quantitative PCR and the $2^{-\Delta\Delta CT}$ Method. *Methods* **2001**, *25* (4), 402–408. https://doi.org/10.1006/meth.2001.1262.

(14) Richman, A.; Swanson, A.; Humphrey, T.; Chapman, R.; McGarvey, B.; Pocs, R.; Brandle, J. Functional Genomics Uncovers Three Glucosyltransferases Involved in the Synthesis of

the Major Sweet Glucosides of Stevia Rebaudiana. *The Plant Journal* **2005**, *41* (1), 56–67. https://doi.org/10.1111/j.1365-313X.2004.02275.x.
(15) Smith, C. A.; Want, E. J.; O'Maille, G.; Abagyan, R.; Siuzdak, G. XCMS: Processing Mass Spectrometry Data for Metabolite Profiling Using Nonlinear Peak Alignment, Matching, and Identification. *Anal. Chem.* **2006**, *78* (3), 779–787. https://doi.org/10.1021/ac051437y.
(16) Tautenhahn, R.; Böttcher, C.; Neumann, S. Highly Sensitive Feature Detection for High Resolution LC/MS. *BMC Bioinformatics* **2008**, *9* (1), 1–16. https://doi.org/10.1186/1471-2105-9-504.
(17) Kuhl, C.; Tautenhahn, R.; Böttcher, C.; Larson, T. R.; Neumann, S. CAMERA: An Integrated Strategy for Compound Spectra Extraction and Annotation of Liquid Chromatography/Mass Spectrometry Data Sets. *Anal Chem* **2012**, *84* (1), 283–289. https://doi.org/10.1021/ac202450g.
(18) van den Berg, R. A.; Hoefsloot, H. C.; Westerhuis, J. A.; Smilde, A. K.; van der Werf, M. J. Centering, Scaling, and Transformations: Improving the Biological Information Content of Metabolomics Data. *BMC Genomics* **2006**, *7* (1), 142. https://doi.org/10.1186/1471-2164-7-142.
(19) Grace, S. C.; Hudson, D. A. *Processing and Visualization of Metabolomics Data Using R*; IntechOpen, 2016. https://doi.org/10.5772/65405.
(20) Pham, T.-N.; Bordage, S.; Pudlo, M.; Demougeot, C.; Thai, K.-M.; Girard-Thernier, C. Cinnamide Derivatives as Mammalian Arginase Inhibitors: Synthesis, Biological Evaluation and Molecular Docking. *International Journal of Molecular Sciences* **2016**, *17* (10), 1656. https://doi.org/10.3390/ijms17101656.
(21) Tanaka, H.; Nakamura, T.; Ichino, K.; Ito, K. A Phenolic Amide from Actinodaphne Longifolia. *Phytochemistry* **1989**, *28* (9), 2516–2517. https://doi.org/10.1016/S0031-9422(00)98022-1.
(22) Hansen, E. H.; Osmani, S. A.; Kristensen, C.; Møller, B. L.; Hansen, J. Substrate Specificities of Family 1 UGTs Gained by Domain Swapping. *Phytochemistry* **2009**, *70* (4), 473–482. https://doi.org/10.1016/j.phytochem.2009.01.013.
(23) Sigrist, C. J. A.; De Castro, E.; Langendijk-Genevaux, P. S.; Le Saux, V.; Bairoch, A.; Hulo, N. ProRule: A New Database Containing Functional and Structural Information on PROSITE Profiles. *Bioinformatics* **2005**, *21* (21), 4060–4066. https://doi.org/10.1093/bioinformatics/bti614.
